# Phytoforensics: Trees as bioindicators of potential indoor exposure via vapor intrusion

**DOI:** 10.1371/journal.pone.0193247

**Published:** 2018-02-16

**Authors:** Jordan L. Wilson, V. A. Samaranayake, Matt A. Limmer, Joel G. Burken

**Affiliations:** 1 U.S. Geological Survey, Missouri Water Science Center, Rolla, Missouri, United States of America; 2 Department of Civil, Environmental, and Architectural Engineering, Missouri University of Science and Technology, Rolla, Missouri, United States of America; 3 Department of Mathematics and Statistics, Missouri University of Science and Technology, Rolla, Missouri, United States of America; 4 Department of Plant and Soil Science, University of Delaware, Newark, Delaware, United States of America; University of Oregon, UNITED STATES

## Abstract

Human exposure to volatile organic compounds (VOCs) via vapor intrusion (VI) is an emerging public health concern with notable detrimental impacts on public health. Phytoforensics, plant sampling to semi-quantitatively delineate subsurface contamination, provides a potential non-invasive screening approach to detect VI potential, and plant sampling is effective and also time- and cost-efficient. Existing VI assessment methods are time- and resource-intensive, invasive, and require access into residential and commercial buildings to drill holes through basement slabs to install sampling ports or require substantial equipment to install groundwater or soil vapor sampling outside the home. Tree-core samples collected in 2 days at the PCE Southeast Contamination Site in York, Nebraska were analyzed for tetrachloroethene (PCE) and results demonstrated positive correlations with groundwater, soil, soil-gas, sub-slab, and indoor-air samples collected over a 2-year period. Because tree-core samples were not collocated with other samples, interpolated surfaces of PCE concentrations were estimated so that comparisons could be made between pairs of data. Results indicate moderate to high correlation with average indoor-air and sub-slab PCE concentrations over long periods of time (months to years) to an interpolated tree-core PCE concentration surface, with Spearman’s correlation coefficients (ρ) ranging from 0.31 to 0.53 that are comparable to the pairwise correlation between sub-slab and indoor-air PCE concentrations (ρ = 0.55, n = 89). Strong correlations between soil-gas, sub-slab, and indoor-air PCE concentrations and an interpolated tree-core PCE concentration surface indicate that trees are valid indicators of potential VI and human exposure to subsurface environment pollutants. The rapid and non-invasive nature of tree sampling are notable advantages: even with less than 60 trees in the vicinity of the source area, roughly 12 hours of tree-core sampling with minimal equipment at the PCE Southeast Contamination Site was sufficient to delineate vapor intrusion potential in the study area and offered comparable delineation to traditional sub-slab sampling performed at 140 properties over a period of approximately 2 years.

## Introduction

**Fig 1:**
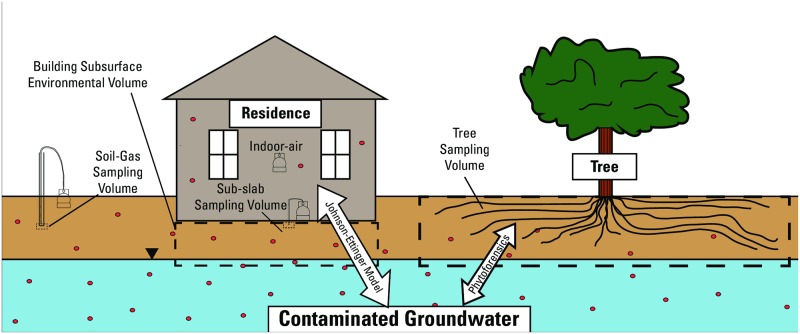
Schematic of the interplay between vapor intrusion, the built environment, and phytoforensic processes.

Vapor intrusion (VI) of volatile organic compounds (VOCs) in the built environment is a threat to human health through migration of carcinogenic contaminants into cracks, seams, and gaps in structures (Fig 1). Although VI can occur in commercial, industrial, or residential settings, residential areas pose unique problems as occupants are unknowingly exposed to concentrations of contaminants in indoor air for long periods, which have a notably greater impact (up to three orders of magnitude more) on human health than outdoor sources [1]. Within the U.S. Environmental Protection Agency (EPA) Superfund program, the VI pathway has recently (2017) been implemented into the Hazard Ranking System [2], allowing a site to be listed on the National Priorities List (NPL) solely because of VI. Because of this increased emphasis on VI, screening for VI will be required at an increasing rate; however, measurement of VI is not simple and is time-, cost-, and labor-intensive, requiring access agreements to enter homes to conduct testing. Simpler, quicker, and more cost-effective screening methods are needed to effectively assess VI and protect human health.

Because VI is a multimedia concern for compounds that exist in vapor, aqueous, and sorbed phases, several different methods exist to assess VI risk. Vapor intrusion risk is typically measured with direct methods (e.g., indoor-air sampling or sub-slab sampling of soil gas) or indirect methods (e.g., groundwater, soil, or soil-gas sampling). These direct and indirect methods for measurement of VI risk are invasive, time and resource intensive [3, 4], or may not be done at all because of the inability to safely collect samples. The current best practice for assessing VI risk is through the application of the two-decade-old Johnson-Ettinger model [5]. The model makes numerous assumptions and attempts to capture a four-dimensional problem using a one-dimensional model. The model is also based on approximately 20 site-specific variables that are difficult to assess [2].

Trees have the potential to measure VI potential *in situ*. Through photosynthesis, trees use solar energy and water potential gradients between the atmosphere and the subsurface to translocate groundwater and in doing so draw moderately hydrophobic contaminants dissolved in groundwater and in the vapor phase across the root-membrane boundary [6, 7]. Once contaminants are in root xylem tissues, the compounds can move with the transpiration stream to aboveground xylem tissues where they can be readily measured.

Phytoforensics has been shown to be a cost- and time-effective tool for semi-quantitatively delineating VOC contamination in groundwater [8–11] and soil vapor [12]. To correlate concentrations in aboveground tree tissue with subsurface contaminant concentrations, phytoforensic methods intercept contaminants as they transport up the xylem and diffuse radially out of the trunk [13]. Phytoforensics uses established trees in the vicinity of contaminant plumes and avoids the time and cost associated with drilling or sample-port installation for sub-slab sampling. Collection of tree-core samples can be conducted in less than five minutes per sample by a single person. Aside from the simplicity, speed, and cost-effective nature of phytoforensics, trees are thought to average subsurface contaminant concentrations by sampling over large subsurface volumes [14, 15] and long time-scales [16, 17], both of which are likely species dependent.

The objective of this work was to assess the correlations between tree-core contaminant concentrations and groundwater, soil, soil-gas, sub-slab, and indoor air contaminant concentrations at a field site. The more traditional matrices were sampled iteratively, allowing comparison with phytoscreening data across multiple sampling times.

## Methods

**Fig 2:**
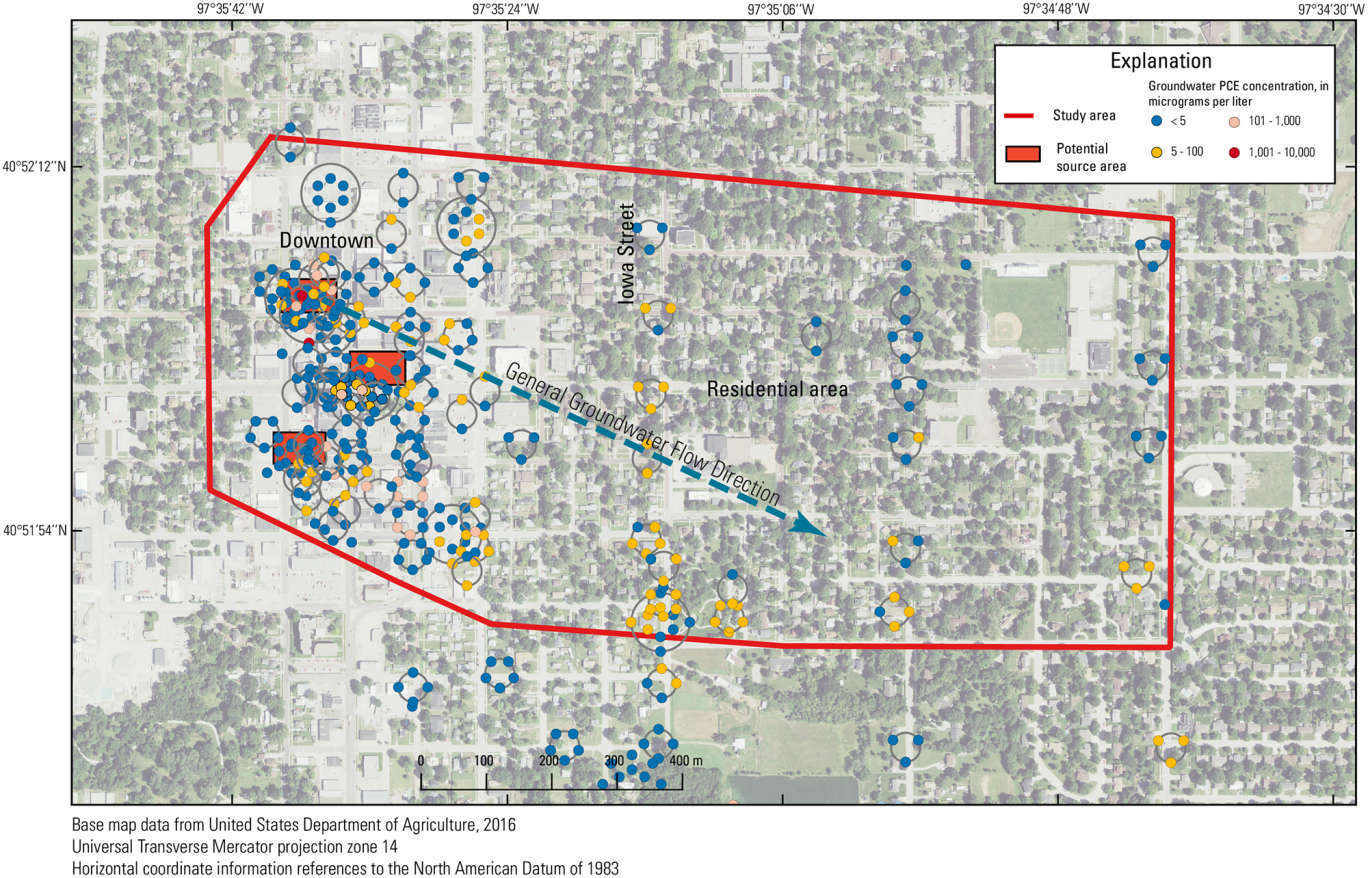
Groundwater tetrachloroethene (PCE) concentrations in the study area in York, Nebraska, from August 2011 to September 2016. Each set of points in concentric rings represents multiple samples in one area.

**Fig 3:**
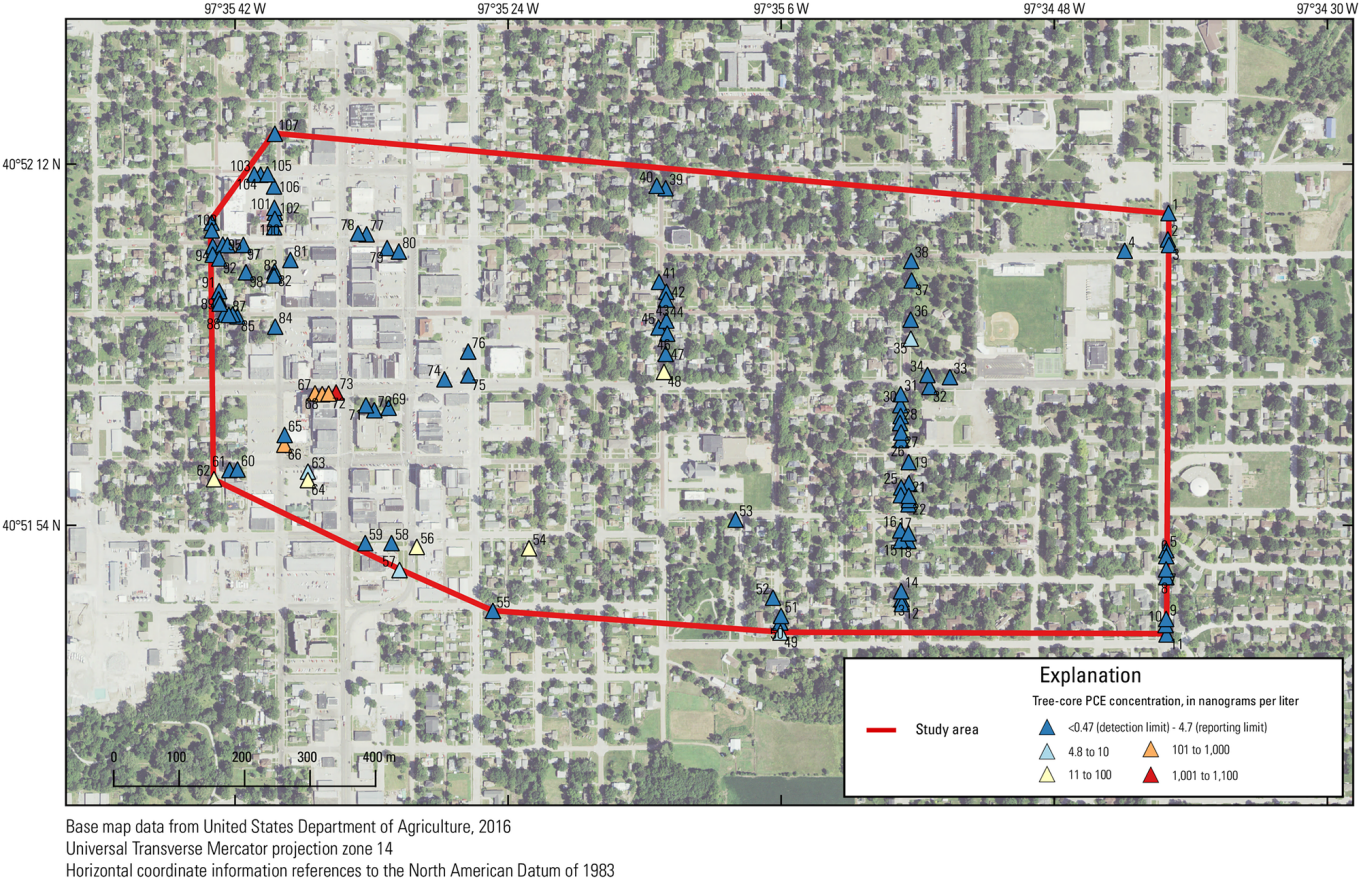
Tree-core tetrachloroethene (PCE) concentrations and corresponding tree numbers in the study area in York, Nebraska, November, 2016.

**Fig 4:**
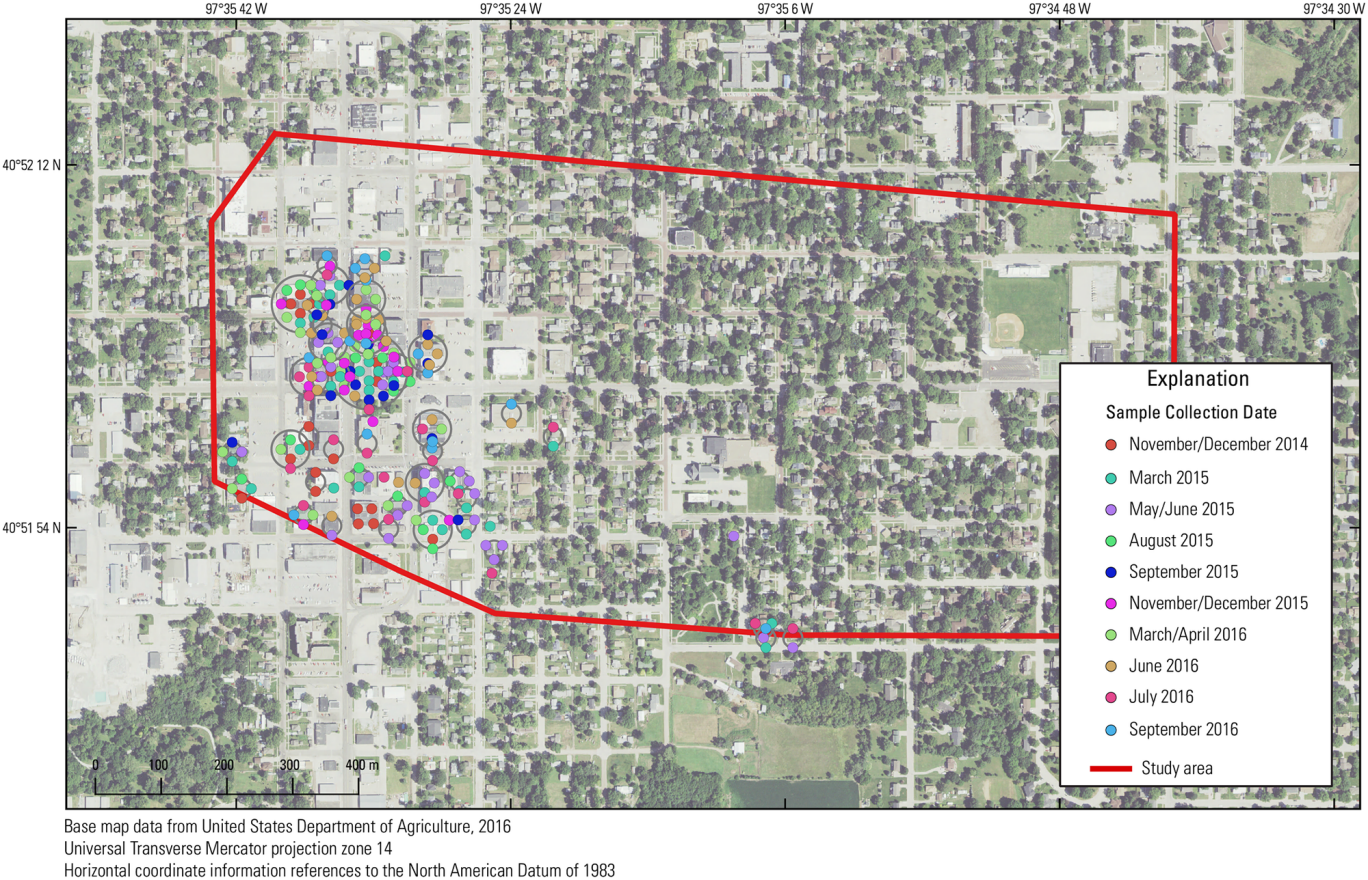
Location and date of indoor-air samples in the study area in York, Nebraska. Each set of points in concentric rings represents multiple samples in one area.

To elucidate the potential of trees to be indicators of VI potential in this study, tree-core samples were collected at the Tetrachloroethene (PCE) Southeast Contamination Site in York, Nebraska (EPA ID NEN000706200), a Superfund site contaminated primarily with PCE. The PCE Southeast Contamination Site was originally listed on the National Priorities List in May 2014 after PCE and other VOCs were detected in private drinking water wells above the EPA maximum contaminant level (MCL). Although the PCE Southeast Contamination Site covers approximately 15 km^2^, the study area for this work covered approximately 2.2 km^2^ encompassing downtown York and the residential area to the east and south (Figs 2 and 3). The potential sources of PCE contamination are from several former dry cleaning businesses in the downtown York area (Fig 2). During the site assessment, the EPA collected groundwater (Fig 2), soil (S4 Fig), indoor-air (Fig 4), and sub-slab (S1 Fig) samples from the commercial and residential properties in the area of the suspected plume. Residential areas surround the downtown area and extend primarily to the north and east.

The underlying geology of the site primarily consists of alluvial deposits. In order of most recent to oldest deposits, the site geology consists of approximately 6 m of clay, 23 m of sand and gravel, 12 m of clay and silt, 15 m of sand, 15 m of clay and silt, and 12 m of sand underlain by Cretaceous age Carlile Shale bedrock. Typical groundwater depths in the area range from 9 m below ground surface (bgs) in the downtown area to about 18 m bgs near the eastern boundary of the study area (Fig 2). The groundwater PCE plume originates in the downtown area and extends to the southeast, the predominant direction of groundwater flow, within the 23 m thick sand and gravel layer.

**Fig 5:**
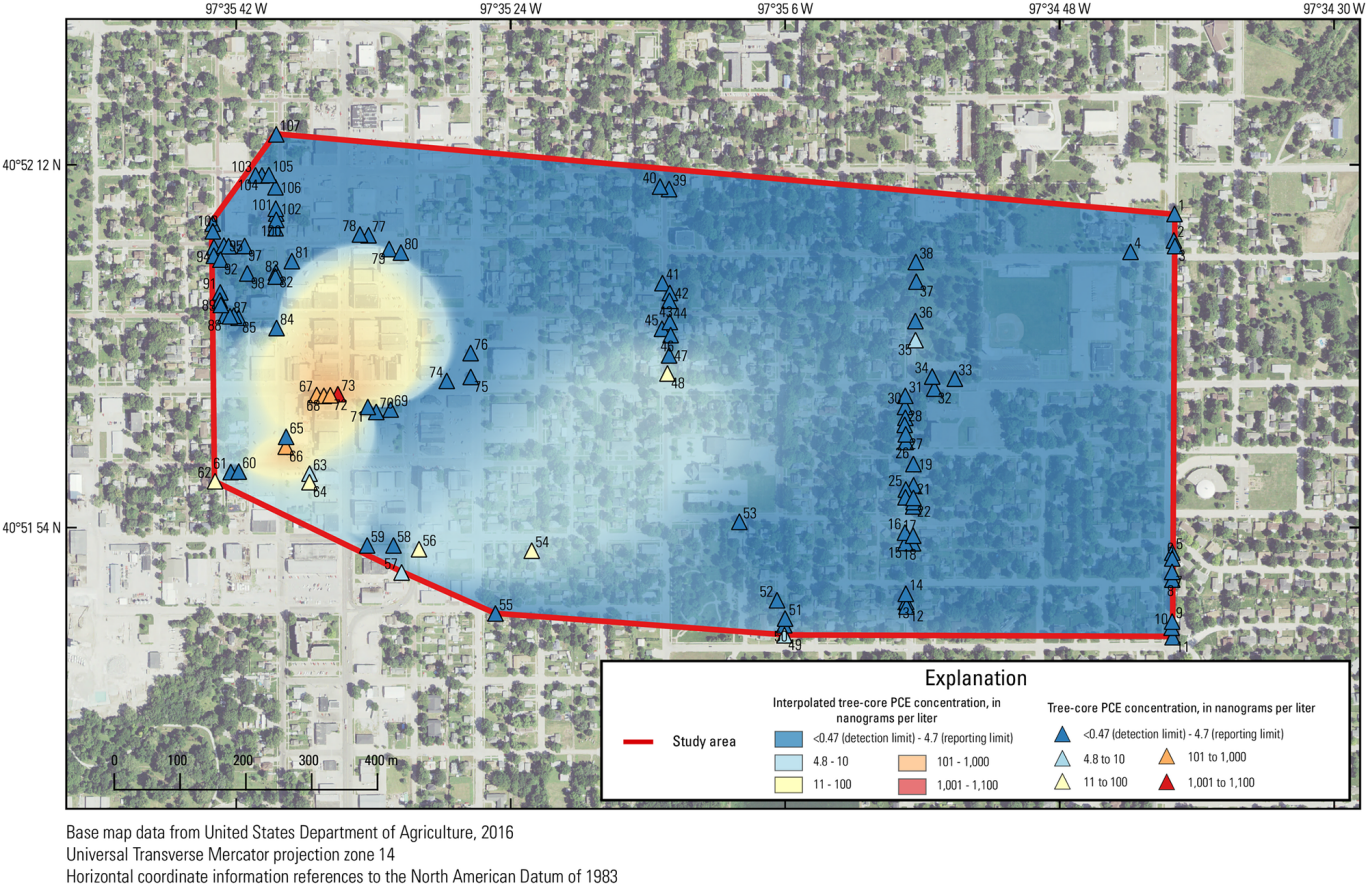
Tree-core tetrachloroethene (PCE) concentrations in the study area overlain on the interpolated tree-core PCE concentration surface.

**Fig 6:**
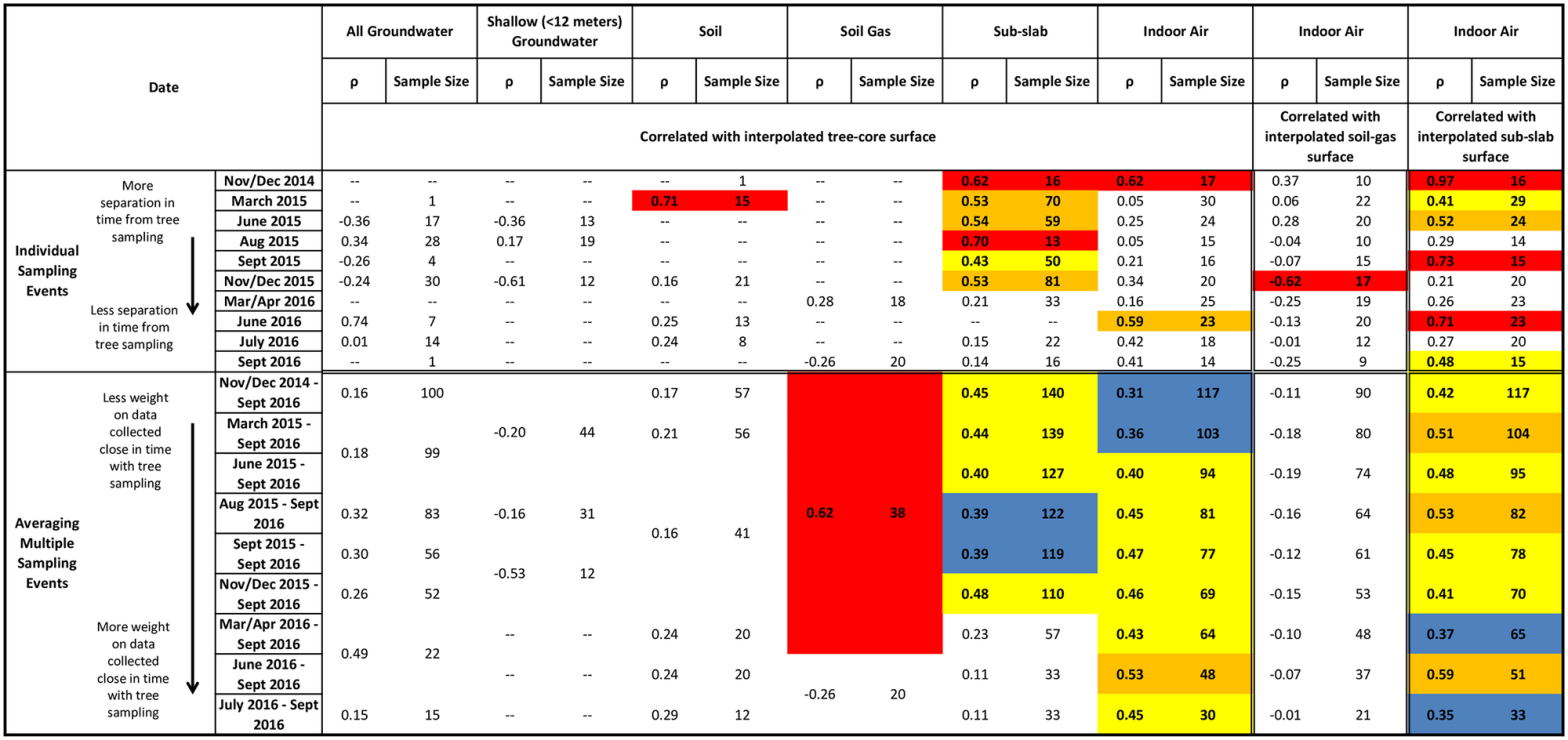
Measures of correlation between groundwater, shallow (<12 m) groundwater, soil, soil-gas, sub-slab, and indoor-air PCE concentrations and interpolated tree-core, soil-gas, and sub-slab PCE concentration surfaces. Cells are colored orange and red if correlation coefficients are 0.50–0.59, and >0.59, respectively, cells are colored blue and yellow if correlation coefficients are 0.30–0.39 and 0.40–0.49, respectively.

In this study, a subset of EPA groundwater, soil, soil-gas, and VI (sub-slab and indoor-air) data was correlated with tree-core samples collected by the USGS to elucidate the potential of trees to be indicators of VI potential. Groundwater, soil, soil-gas, and VI samples were collected by EPA Region 7 from January 2010 to September 2016, and these data underwent typical EPA quality-assurance/quality-control procedures. Within the study area, EPA collected samples over 10 separate sampling events from November 2014 to September 2016. Tree-core samples were collected at the PCE Southeast Contamination Site in York, Nebraska during November 2–3, 2016 (Figs 3 and 5). Because trees are thought to provide information about subsurface VOC concentrations over a period of time, tree-core PCE concentrations collected in November 2016 were used to develop correlations with EPA data from each of the 10 sampling events as well as with aggregations of EPA data ranging from the aggregation of all sampling events from November 2014 to September 2016 period to the aggregation of the last two sampling events (July 2016 and September 2016; Fig 6). Correlations were then compared between individual sampling events as well as over periods of time to determine over what period of time tree-core PCE data is informative of subsurface contamination and, more specifically, vapor intrusion. All data used in this paper are available at U.S. Geological Survey (USGS) ScienceBase, https://doi.org/10.5066/F7CF9P06.

### Tree-core sampling

Tree-core samples were collected at the site from the south side of trees by a two-person team over 12 hours during November 2–3, 2016, using published tree-coring methods [18]. A total of 121 samples, which included 109 tree-core samples, 10 replicate samples collected 1–3 cm above the tree-core sample, 1 trip-blank sample, and 1 field-blank sample, were collected. A total of 53 trees, ranging from 11.4 to 142 cm in diameter, were sampled in the primarily residential area east of Iowa Street and the remaining 56 trees were sampled in the vicinity of the downtown York area (Fig 3). It has been shown that contaminant concentrations in tree-core samples vary between tree species [18, 19], and dominant tree genera (and sample number, *n*) at the site included oak (*Quercus* sp.; *n* = 27), maple (*Acer* sp.; *n* = 20), elm (*Ulmaceae* sp.; *n* = 17), ash (*Fraxinus* sp.; *n* = 10), Bradford pear (*Pyrus* sp.; *n* = 10), crab apple (*Malus* sp.; *n* = 10), ginkgo (*Ginkgo biloba*; *n* = 4). Tree-core samples were collected using a 0.25-inch diameter by 12-inch long steel increment borer and a standard 0.2-inch diameter core sample (3 inches long) was extracted from each tree at a 1-m height. Tree-core samples were analyzed at the Environmental Research Center at the Missouri University of Science and Technology in Rolla, Missouri using published methods [11]. Tree-core samples were allowed to reach equilibrium overnight and run on an Agilent 7890 gas chromatograph (Agilent Technologies, Inc., Santa Clara, California) with a micro-electron-capture detector fitted with a CombiPAL solid-phase microextraction (SPME) fiber autosampler. Samples were mass-corrected and PCE concentrations were reported in nanograms of contaminant per liter of sap (ng/L) using published methods [11]. Global positioning system (GPS) locations were collected for each tree using a Trimble GeoExplorer XH^®^ (Trimble Navigation Limited, Sunnyvale, California) with a sub-meter accuracy.

Although tree density in the downtown area was low, most trees were sampled. In contrast, tree density in the residential area was high, but samples were only collected along a few streets in the residential area. Both sources of data sparsity left large spatial gaps in the tree-core dataset. Because the source area and many of the EPA samples were located in the downtown area where tree-core data were relatively sparse, the spatial distribution of the tree-core PCE concentration surface may not be fully defined in that area, which could negatively affect correlations between EPA data and the tree-core PCE concentration surface.

### Groundwater, soil, and soil-gas sampling

A total of 1,198 groundwater samples were collected and analyzed by the EPA from January 2010 to September, 2016 from the site. Of the 629 samples collected between November 2014 and September 2016 (S8 Fig), 371 samples were contained within the region from where tree-core samples were collected (the “study area”). All 371 samples were collected between August 2011 and September 2016 (Fig 2) using temporary direct-push wells using a Geoprobe Screen Point 16 (Geoprobe, Salina, Kansas) apparatus containing a reusable stainless steel screen. Samples were collected between 9 and 40 m bgs (average of approximately 15 m) in 40-mL vials. Samples were collected from multiple depths within each well with at least one sample near the top of the water table. Correlations were developed with all groundwater data as well as only the shallowest groundwater samples within each well and < 12 m deep bgs (S6 Fig) because VOC concentrations in trees have been shown to be better correlated with VOC concentrations in the shallow subsurface [12].

A total of 209 soil samples were collected and analyzed by the EPA from November 2011 to July 2016 from the study area, and 188 samples were collected in the downtown area. Of the 209 samples, 57 samples were collected between November 2014 and September 2016 (S4 Fig) from depths ranging from 0 to 27 m bgs using direct-push technology using a Macro-Core sampler fitted with a disposable polyvinyl chloride liner according to EPA standard operating procedure.[20] At a given interval selected for sampling, a tipless syringe was used to collect five grams of soil, which was transferred into a 40-mL vial for analysis by the EPA Region 7 laboratory.

EPA collected 18 soil-gas samples in April 2016 in the downtown area and collected 20 soil-gas samples in September 2016 in the residential area to the east and southeast (S2 Fig). All samples were collected using established soil-gas methods with a Geoprobe^®^ Post Run Tubing (PRT) soil-gas sampling system. Samples were collected in Tedlar^®^ bags as a discrete concentration (30- to 60-second time-weighted average [TWA]) at a flowrate of about 5 L/min. All samples were analyzed onsite by the EPA Region 7 mobile laboratory with gas chromatography-mass spectrometry (GC-MS).

### VI sampling

A total of 255 indoor-air samples (Fig 4) and 461 sub-slab samples (S1 Fig) were collected by the EPA in residential and commercial buildings between July 2014 and September 2016. Indoor-air concentrations were determined using TWA concentration over about 20 hours obtained from SUMMA^®^ canisters deployed at the lowest level inside each building. Sub-slab concentrations were determined either as a TWA using SUMMA^®^ canisters or as a discrete concentration (30- to 60-second TWA) using Tedlar^®^ bags at a flowrate of about 5 L/min. Discrete samples were first collected to screen all locations once ports were installed, and SUMMA^®^ canisters were later used to collect TWAs. Although these two types of samples represent concentrations over different periods of time, for the purposes of this paper, these data are treated equal because the difference in time spans are negligible compared to the total time span of the dataset. All samples were analyzed by the EPA Region 7 laboratory with GC-MS.

### Data analysis

Because tree-core samples were not collocated with groundwater, soil, soil-gas, sub-slab, and indoor-air samples, interpolated surfaces for tree-core, soil-gas, and sub-slab PCE concentrations were estimated so that comparisons could be made between pairs of data. Interpolated surfaces were estimated using a conservative approach by creating a triangulated irregular network (TIN) from the concentration data and interpolating a 1-m raster surface from the TIN using natural neighbors interpolation [21, 22] in ArcMap ^®^ (Environmental Systems Research Institute, Redlands, California) and the Python (Python Software Foundation, Delaware) module ArcPy. In addition to being the most conservative interpolation method, the TIN method resulted in the most hydrogeologically realistic surface compared to surfaces developed using kriging and inverse-distance-weighting methods. Mean soil-gas and sub-slab PCE concentrations at each location were also interpolated to calculate correlation with indoor-air PCE concentrations. Because of the sparsity of trees in the downtown area, the spatial distribution of tree-core PCE concentration data was non-uniform; therefore, the interpolated tree-core PCE concentration surface may not be fully defined in that area, which could affect correlations between EPA data and the interpolated tree-core PCE concentration surface. To evaluate error in defining the tree-core concentration surface in areas distant from sampled trees, additional tree-core samples would need to be collected in the area of interest and compared to the estimated surface.

To correlate groundwater, soil, soil-gas, sub-slab, and indoor-air PCE concentrations with the interpolated tree-core PCE concentration surface and to correlate indoor-air PCE concentrations with the interpolated soil-gas and sub-slab PCE concentration surfaces, the nonparametric Spearman’s rank correlation coefficient [23] (ρ) was used at a significance level of 0.05. In this paper, low, moderate, and high values of ρ are defined as less than (<) 0.3, between 0.3 and 0.5, and greater than (>) 0.5. To develop the correlation dataset, PCE concentrations were extracted from the interpolated tree-core, soil-gas, and sub-slab surfaces at each groundwater, soil, soil-gas, sub-slab, or indoor-air sample location. Correlations were calculated for each of the 10 sampling events as well as over multiple sampling events because trees are thought to provide information about subsurface concentrations over a period of time. For correlations over multiple sampling events and with multiple samples from one location on any one sampling event, averages were calculated and used in correlation analysis. Because there is little spatial variability between sub-slab and indoor-air sample locations, correlations between paired sub-slab and indoor-air samples collected during the same sampling event were also calculated.

To assess the effect of the non-uniform distribution of samples on the error in the interpolated tree-core PCE surface, groundwater, soil, soil-gas, sub-slab, and indoor-air PCE concentrations were also correlated only with tree-core PCE concentrations in tree-core samples located within 31 m. These correlations minimize the potential error that may be introduced with non-uniformly distributed tree-core samples and are most representative of the true correlation between tree-core concentrations and nearby vapor intrusion concentrations.

## Results and discussion

### Trees as indicators

Concentrations of PCE in tree-core samples from the 109 trees sampled were above the detection limit (0.47 ng/L) [11] in 37 trees and above the reporting limit (4.7 ng/L) in 14 trees. Of the 14 trees with PCE concentrations above the reporting limit, 11 were in the vicinity of the downtown area with concentrations as high as 1,100 ng/L (Fig 5). Concentrations of PCE in tree-core samples were also relatively large (250–500 ng/L) in samples east and southeast of the downtown area along the direction of groundwater flow (Fig 2). Concentrations of PCE were all below detection in all blanks, and the relative percent difference in duplicates ranged from 7.2 to 12.2% (S1 Table), indicating that tree-core results at the site were highly reproducible. Although contaminant concentrations change seasonally depending because of changes in transpiration [24], concentrations likely decrease less than an order of magnitude and the relative distribution of concentrations likely is not substantially changed when sampling in November.

#### Groundwater

Because groundwater PCE concentrations were variable and the distribution of available trees to sample was poor in the downtown area, correlations of groundwater PCE concentrations to the interpolated tree-core PCE concentration surface were poor (Fig 6). When either shallow or all groundwater data were included, no significant correlations existed between groundwater PCE concentrations and the interpolated tree-core PCE concentration surface for any sampling event or period of sampling. Where groundwater PCE concentrations were large, tree-core data were sparse, and as a result, did not allow for full definition of the concentration distribution in those areas (S7 and S9 Figs). Shallow groundwater samples, which were close to the water table surface, had poor correlation with the interpolated tree-core PCE concentration surface. A better assessment of correlation between groundwater PCE and the interpolated tree-core PCE concentration surface would likely result if tree-core samples were more uniformly distributed within the study area. Additionally, many of the groundwater samples were collected greater than 15 m bgs; therefore, trees in some areas might be taking up shallower, infiltrated groundwater from local recharge with smaller PCE concentrations from dilution than the samples at depth. A recent review of 12 sites in a previous study found that the coefficient of determination (R^2^) between tree-core and groundwater concentrations increased from 0.56 to 0.88 when deeper (>4 m) groundwater was excluded [25], suggesting the poor correlation between tree and groundwater contamination at this site may be a result of both data sparsity and depth of groundwater.

#### Soil and soil-gas

Because of the scarcity of tree-core data in areas where soil data was collected (S5 Fig), correlations between soil PCE concentrations and the interpolated tree-core PCE concentration surface were poor (Fig 6). Because tree-core PCE concentrations have been shown to correlate well with soil samples [12, 26], tree-core data sparsity and large local variability of soil samples likely resulted in poor correlation between soil PCE concentrations and the interpolated tree-core PCE concentration surface in this study. There was high correlation (n = 15; ρ = 0.71) when comparing soil samples collected during March 2015 with the interpolated tree-core PCE concentration surface, likely because the soil samples were located in areas with tree-core samples. Subsequent soil sampling events were focused in the downtown area where tree-core samples were not collected. One previous study reported stronger correlations between soil and tree PCE (R^2^>0.8 for soil depths up to 3.6 m) than between groundwater and tree PCE (R^2^ = 0.48, groundwater about 6 m bgs) [12].

Although no significant correlation existed between soil-gas PCE concentrations and the interpolated tree-core PCE concentration surface for individual sample events, combining the two soil-gas sampling events (March/April 2016 and September 2016) resulted in high correlation (n = 38; ρ = 0.62; Fig 6 and S3 Fig). Because the different sampling events were spatially clustered, combining the two sampling events resulted in a more distributed dataset. Investigations comparing tree and soil gas as preliminary site screening tools are limited. One study reported both techniques were able to locate one source area, but only tree coring was able to locate a second source [27].

#### Sub-slab

**Fig 7:**
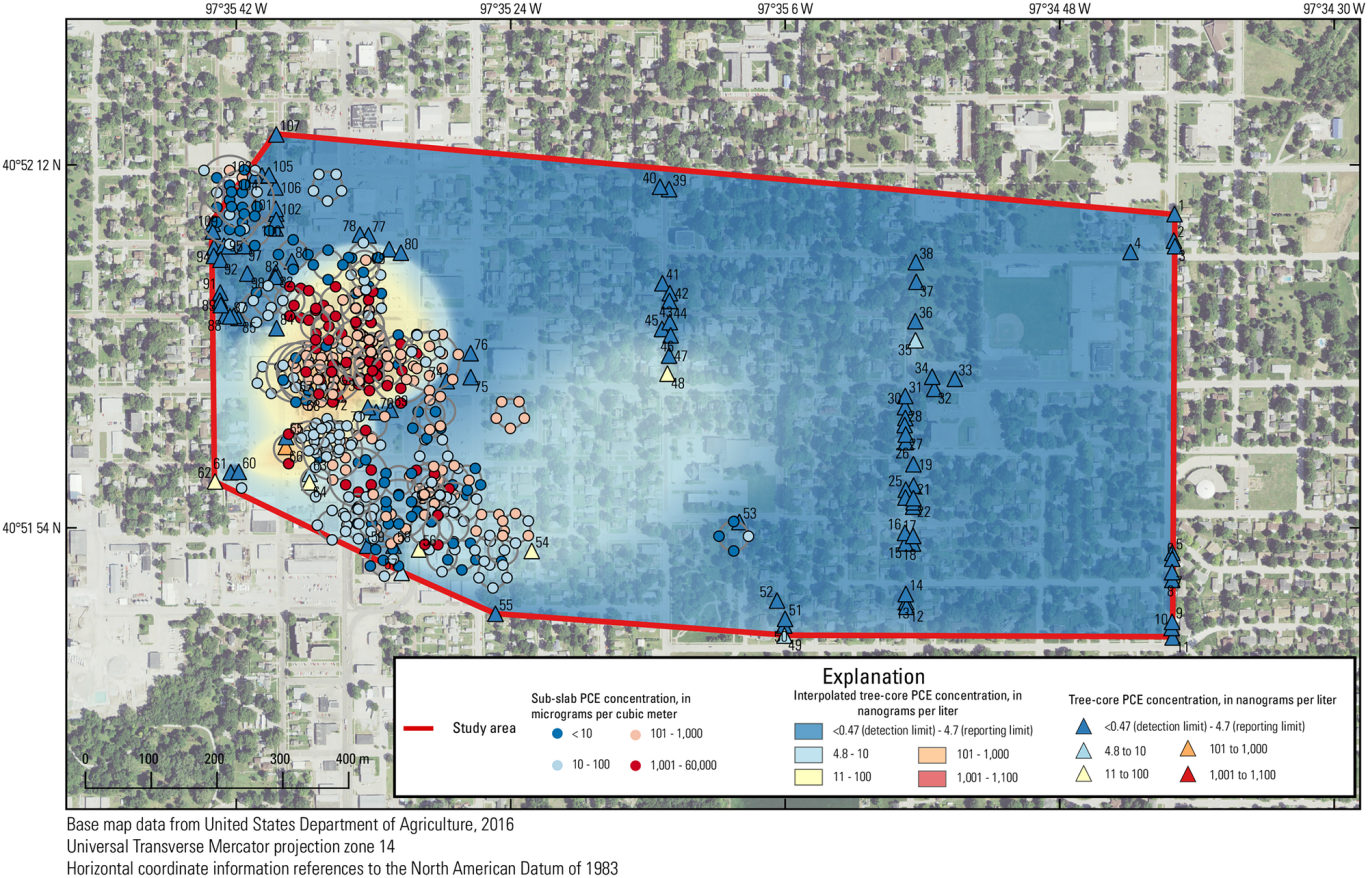
Sub-slab tetrachloroethene (PCE) concentrations in the study area overlain on the interpolated tree-core PCE surface. Each set of points in concentric rings represents multiple samples in one area.

Correlations between sub-slab PCE concentrations and the interpolated tree-core PCE concentration surface were frequently significant, with significant coefficients ranging from 0.43 to 0.70 over all individual sampling events from November 2014 to December 2015 (Figs 6 and 7). Unlike groundwater and soil correlations, correlations between sub-slab PCE concentrations and the interpolated tree-core PCE concentration surface were consistently correlated over time until March/April 2016 and decreased when the majority of the dataset consisted of more recent data from March/April 2016 to September 2016 sampling events that included samples in areas not well defined by the tree-core sample density (S1 Fig).

#### Indoor air

**Fig 8:**
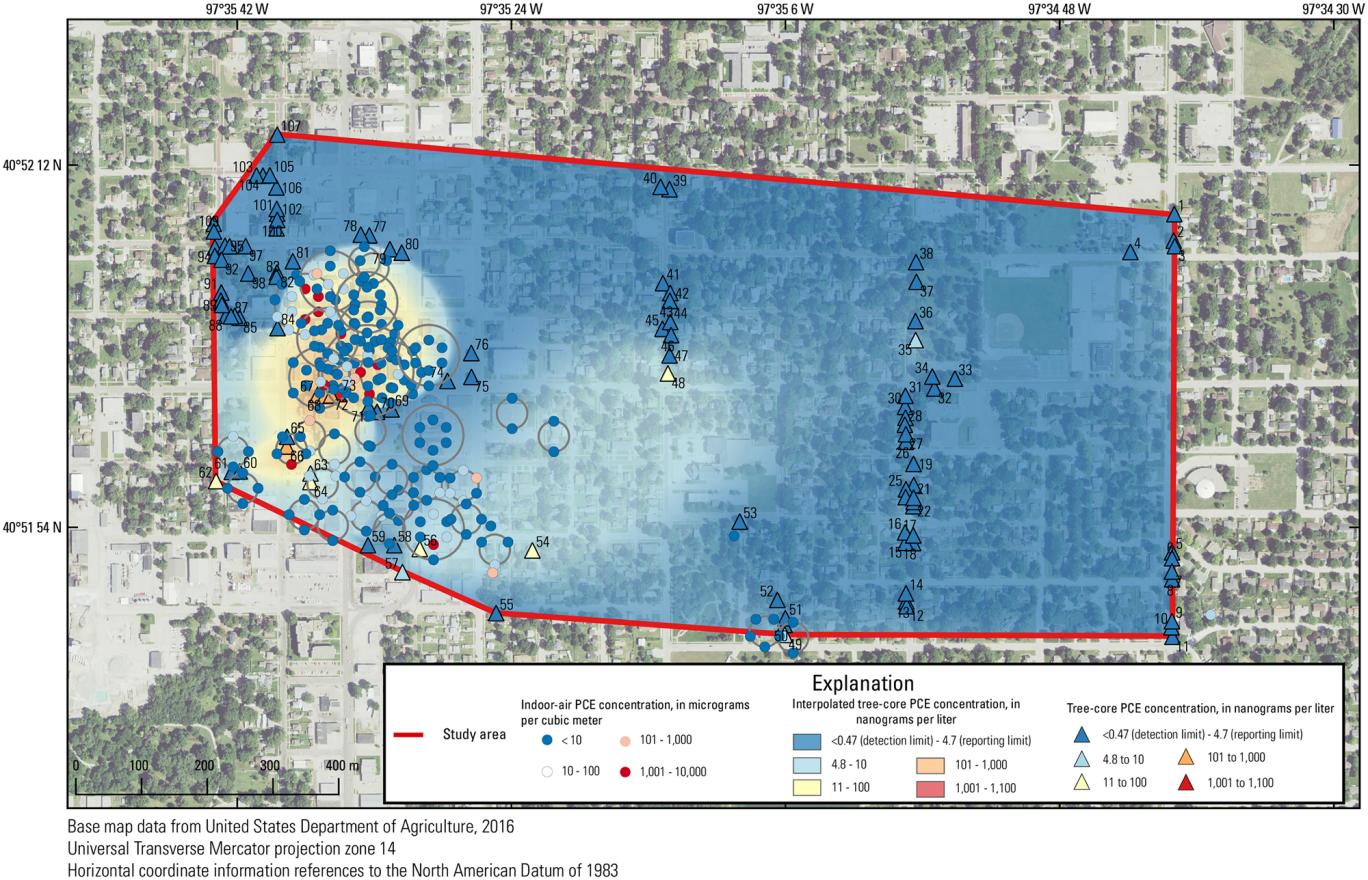
Indoor-air tetrachloroethene (PCE) concentrations in the study area and interpolated tree-core PCE concentration surface. Each set of points in concentric rings represents multiple samples in one area.

Correlation coefficients between indoor-air PCE concentrations and the interpolated tree-core PCE concentration surface were high for the November/December 2014 (ρ = 0.62, n = 17) and June 2016 (ρ = 0.59, n = 23) individual sampling events, but were not significant for all other individual sampling events (Figs 6 and 8). However, when correlating groups of averaged indoor-air sampling events to the interpolated tree-core PCE concentration surface, correlations were significant for all sampling periods. Correlation coefficients ranged from 0.31 to 0.53 and were generally higher for more recent sampling periods. Because aggregating multiple sampling events averages samples from the same location and increases the number of sampled locations through longer time periods, consistently significant and moderate correlations could indicate that the average indoor-air PCE concentrations, rather than the individual indoor-air PCE concentration, are more correlated to the interpolated tree-core PCE surface (i.e., tree sampling is more indicative of a TWA than a “snapshot”), especially since the relative standard deviation of indoor-air PCE concentrations is large (5.18). The improvement of correlation also could be the result of larger and better distributed sample sizes.

### Soil-gas as an indicator of indoor air

Correlations between indoor-air PCE concentrations and the interpolated soil-gas PCE concentration surface (produced from data from two sampling events) for individual sampling events and aggregated sampling events were poor with only one significant correlation coefficient of -0.62, which occurred during the November/December 2015 sampling event (Fig 6 and S10 Fig). Because soil-gas samples were collected along two city streets, they suffer from the same effects of poor spatial distribution of samples that is observed in the tree-core dataset.

### Sub-slab as an indicator of indoor air

**Fig 9:**
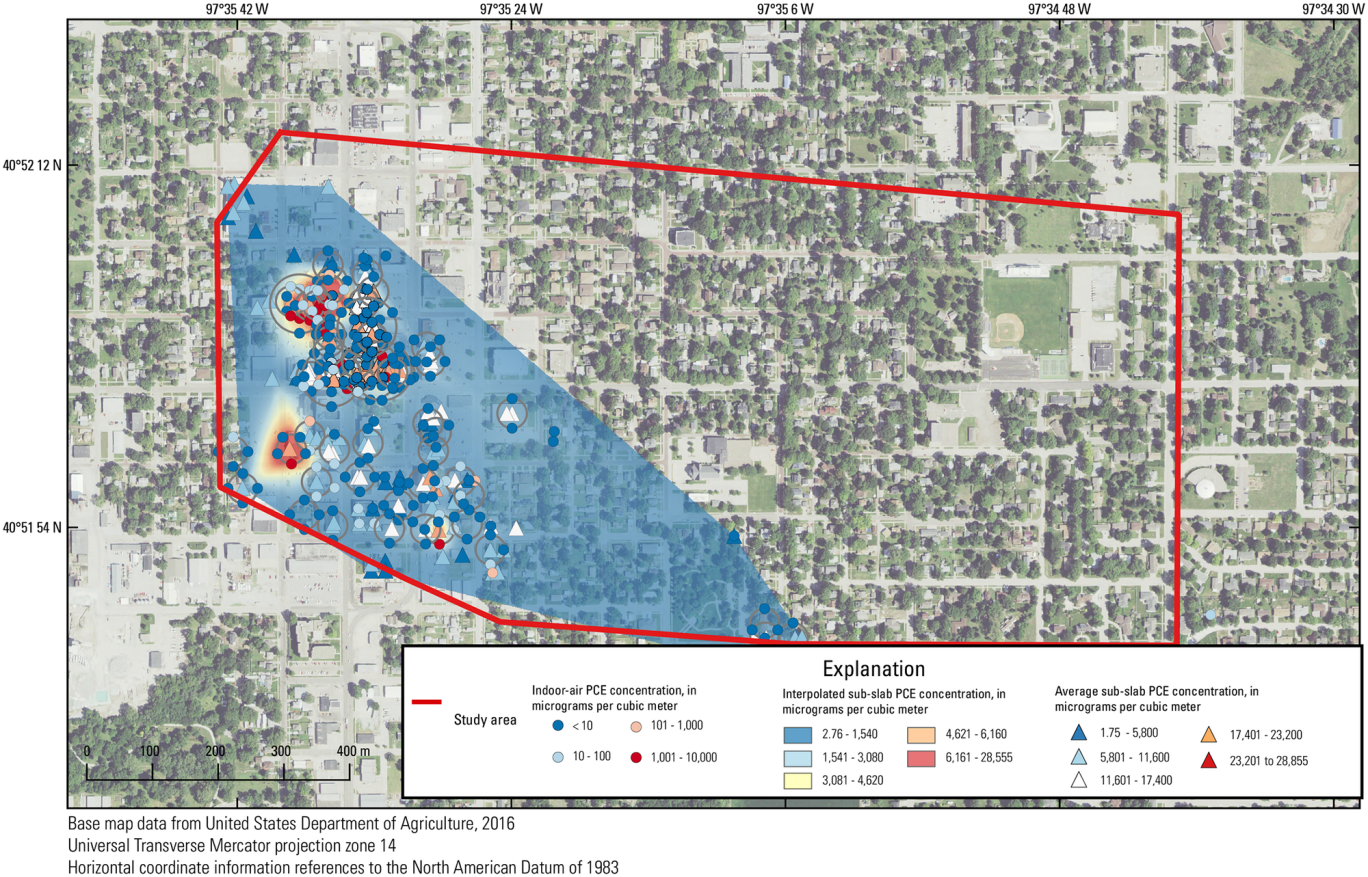
Indoor-air tetrachloroethene (PCE) concentrations in the study area overlain on the interpolated sub-slab PCE concentration surface. Points in each concentric ring represent multiple sampling events at one location.

Correlation coefficients between indoor-air PCE concentrations and the interpolated average sub-slab PCE concentration surface (from multiple sampling events) were substantially higher than those between indoor-air PCE concentrations and the interpolated soil-gas PCE concentration surface (Figs 6 and 9). A total of 6 out of 10 individual sampling events had moderate to high significant correlations. Significant correlation coefficients ranged from 0.41 to 0.97, with the 0.97 coefficient occurring during the November/December 2014 sampling event, and no general trend with time was observed.

Because one of the current best available methods for screening for vapor intrusion risk is sub-slab sampling, a pairwise correlation test was conducted on all paired indoor-air and sub-slab samples collected during the same sampling event and location and indicated a significant correlation coefficient of 0.55 (n = 89), which is comparable to the correlation between indoor-air and the interpolated tree-core PCE concentration surface.

### Effect of non-uniform tree-core sample distribution on correlations

**Fig 10:**
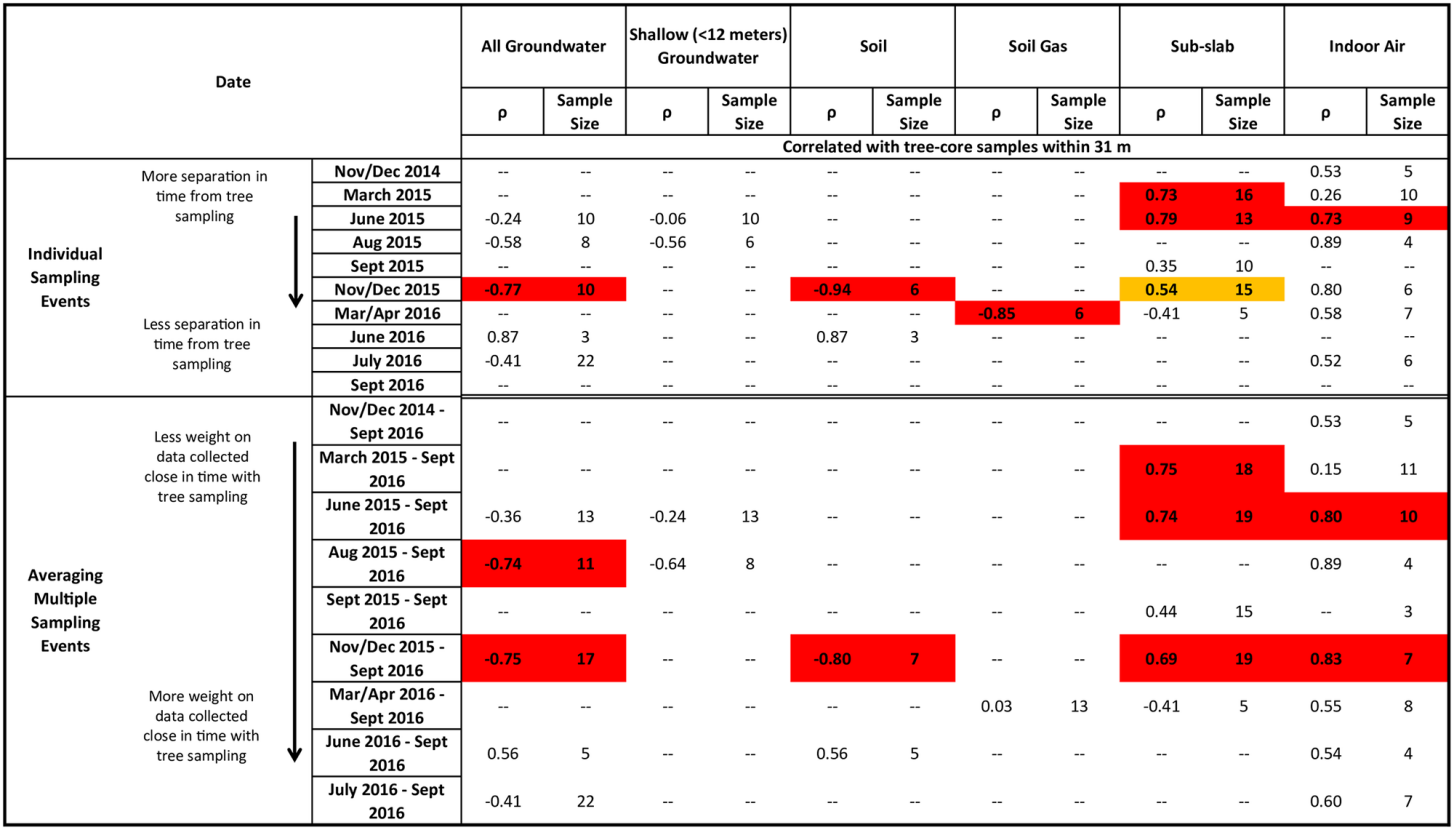
Measures of correlation between groundwater, shallow (<12 m) groundwater, soil, soil-gas, sub-slab, and indoor-air PCE concentrations and tree-core concentrations in tree-core samples collected within 31 m. Cell highlighted with color with bold font are significantly correlated. Cells are colored orange and red if correlation coefficients are 0.50–0.59, and >0.59, respectively.

Correlations between indoor-air and sub-slab PCE concentrations and tree-core samples collected within 31 m (Fig 10) generally agreed with the correlations between indoor-air and sub-slab PCE concentrations and the interpolated tree-core PCE surface (Fig 6 and S11 Fig). All significant correlation coefficients were moderate or high with values ranging from 0.54 to 0.83. Although correlations were insignificant for many individual sampling events and sampling periods, correlation significance is limited in part by small sample sizes. In contrast, significant correlations between groundwater, soil, and soil-gas PCE concentrations and tree-core samples collected within 31 m (Fig 10) were negatively correlated with high values ranging from -0.74 to -0.94. This is likely explained by the large amount of heterogeneity in the subsurface over small distances, especially near the source areas downtown where most of the groundwater, soil, and soil-gas samples in these correlations are located. Future work should be focused on collecting tree-core samples closely paired with vapor intrusion samples in order to better evaluate correlations between vapor intrusion and tree-core samples without the potential error associated with non-uniform distributions of tree-core samples. The use of a diameter-dependent buffer may also be useful in correlations as it has been shown that trees represent a subsurface volume proportionate to their trunk diameter [28].

## Conclusions and site implications

As VI transport involves multi-media environmental transport and the heterogeneities inherent in urban environments, multiple sampling approaches of various media are commonly applied, such as groundwater, soil-gas, sub-slab, and indoor-air. Because tree roots interact with subsurface water, soil, and vapor, phytoforensic sampling has been shown to offer a blending or composite of these multiple environmental media [12, 26, 28]. Findings shown here indicate that PCE concentrations in trees can be highly correlated with multiple VI investigative methods, including soil-gas and VI samples (sub-slab and indoor-air samples), especially when comparing to indoor-air concentrations over long periods, and are comparable to sub-slab samples. Indoor-air PCE concentrations in this study collected within 2 months and, to a lesser extent, with average indoor-air concentrations over 2 years, were highly correlated with the interpolated tree-core PCE concentration surface. Although this is only one case study, these findings and previous studies [28, 29] suggest that trees are valid indicators of VI potential over long temporal periods.

The non-uniform distribution of tree-core samples in this specific study left spatial gaps in the dataset, especially in the residential area, and likely resulted in poor definition of the tree-core PCE concentration surface. Collection of tree-core samples should be in a more uniformly distributed pattern over the study area to avoid spatial gaps in tree-core data and to better define the tree-core PCE concentration distribution. When non-uniform distributions for tree sampling are unavoidable due to the sparsity of available trees, more confidence should be attributed to tree-core data proximal to the area of interest. Because of this inverse relationship between confidence and distance, trees should be more densely sampled in the vicinity of receptors in order to provide the most accurate estimates of VI potential.

Collecting uniformly distributed tree-core data of sufficient density over the entire project area is vital to fully describe the tree-core concentration surface. Prior knowledge of the local hydrogeology and potential source areas can also be used to direct sample density in certain areas as tree-core samples can be collected in greater densities in the vicinity of the source areas with decreasing spatial density in the downgradient direction. This approach is relatively easy to accomplish, because tree-coring is an exceptionally rapid and inexpensive practice compared to traditional methods. With each sample taking less than 5 minutes to collect, a large study area on the order of square kilometers of can be sampled in days compared to the weeks and months required from traditional methods. In this study of approximately 1 km^2^ with less than 60 trees in the vicinity of the source area, roughly 12 hours of tree-core sampling with minimal equipment was sufficient to delineate the vapor intrusion potential and offered comparable delineation to traditional sub-slab sampling performed at 140 properties over a period of approximately 2 years with multiple mobilizations of large, vehicle-mounted equipment.

At this site, phytoscreening would have been suitable as an initial screening tool to determine the general areas of interest to focus future VI sampling. Because of the cost- and time-efficient nature of tree-coring, several hundred tree-core samples can be collected in a week’s time depending on the required permissions on individual properties (often one of the limiting factors in phytoforensic studies); however, if trees are located in a city right-of-way and permission is granted by the city, tree-core samples can be collected simply by walking along city streets with less than 5 minutes per sample required. Alternatively, phytoscreening could have been performed iteratively by performing an initial, sparse sampling, followed by denser sampling in areas of interest. In areas of interest with limited trees, soil-gas or other screening techniques would be necessary. Ideally, such a sampling plan would minimize the number of sub-slab and/or indoor-air samples collected.

## Supporting information

S1 Fig Location and date of sub-slab samples in the York, Nebraska study area. Each set of points in concentric rings represents multiple samples in one area.
(TIF)

S2 Fig Location and date of soil-gas samples in the York, Nebraska study area. Each set of points in concentric rings represents multiple samples in one area.
(TIF)

S3 Fig Soil-gas tetrachloroethene (PCE) concentrations in the study area overlain on the interpolated tree-core PCE concentration surface. Each set of points in concentric rings represents multiple samples in one area.
(TIF)

S4 Fig Location and date of soil samples in the York, Nebraska study area. Each set of points in concentric rings represents multiple samples in one area.
(TIF)

S5 Fig Soil tetrachloroethene (PCE) concentrations in the study area overlain on the interpolated tree-core PCE concentration surface. Each set of points in concentric rings represents multiple samples in one area.
(TIF)

S6 Fig Location and date of shallow (<12 m) groundwater samples, York, Nebraska in the study area. Each set of points in concentric rings represents multiple samples in one area.
(TIF)

S7 Fig Groundwater tetrachloroethene (PCE) concentrations in shallow (< 12 m) groundwater samples in the study area overlain on the interpolated tree-core PCE concentration surface. Each set of points in concentric rings represents multiple samples in one area.
(TIF)

S8 Fig Location and date of all groundwater samples in the York, Nebraska study area. Each set of points in concentric rings represents multiple samples in one area.
(TIF)

S9 Fig Groundwater tetrachloroethene (PCE) concentrations in all groundwater samples in the study area overlain on the interpolated tree-core PCE concentration surface. Each set of points in concentric rings represents multiple samples in one area.
(TIF)

S10 Fig Indoor-air tetrachloroethene (PCE) concentrations in the study area overlain on the interpolated soil-gas PCE concentration surface. Each set of points in concentric rings represents multiple samples in one area.
(TIF)

S11 Fig Average concentrations of tetrachloroethylene (PCE) in sub-slab versus interpolated tree-core surface a) from November 2014 to September 2016 and b) from July 2016 to September 2016 as well as average concentrations of tetrachloroethylene (PCE) in indoor-air samples from June 2016 to September 2016 versus c) the interpolated PCE tree-core surface and d) the interpolated PCE sub-slab surface. Rho is the spearman’s rank correlation coefficient.
(TIF)

S1 Table Quality-assurance results for blanks and duplicates. (TIF)
