# Supplementary figures and images for: Phytoforensics: Trees as bioindicators of potential indoor exposure via vapor intrusion
Source: PLoS One. 2018 Feb 16;13(2):e0193247. doi: 10.1371/journal.pone.0193247 (PMC5815607; doi:10.1371/journal.pone.0193247)

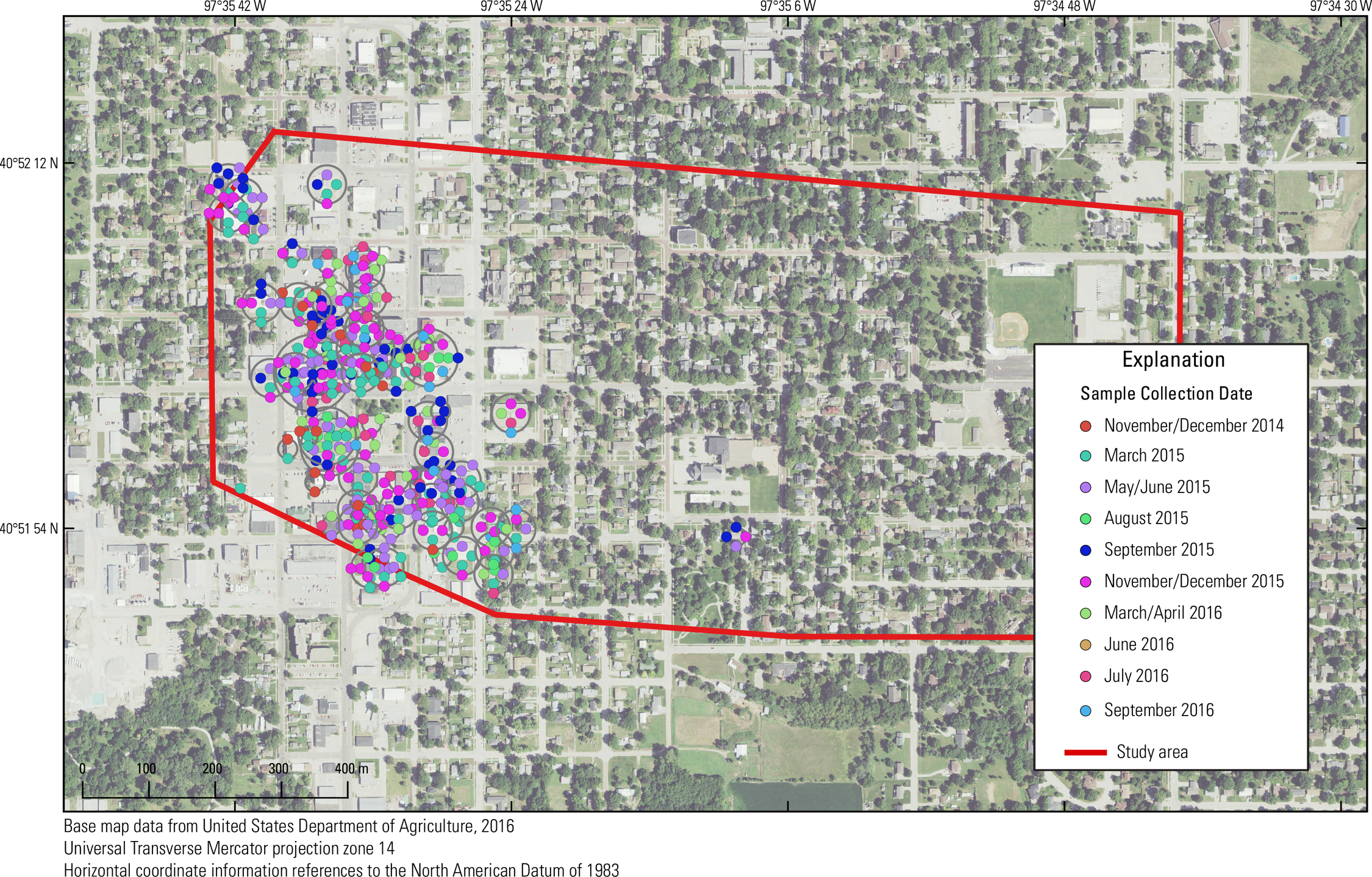

Supplement: S1 Fig — Each set of points in concentric rings represents multiple samples in one area. (TIF) [file pone.0193247.s001.tif]

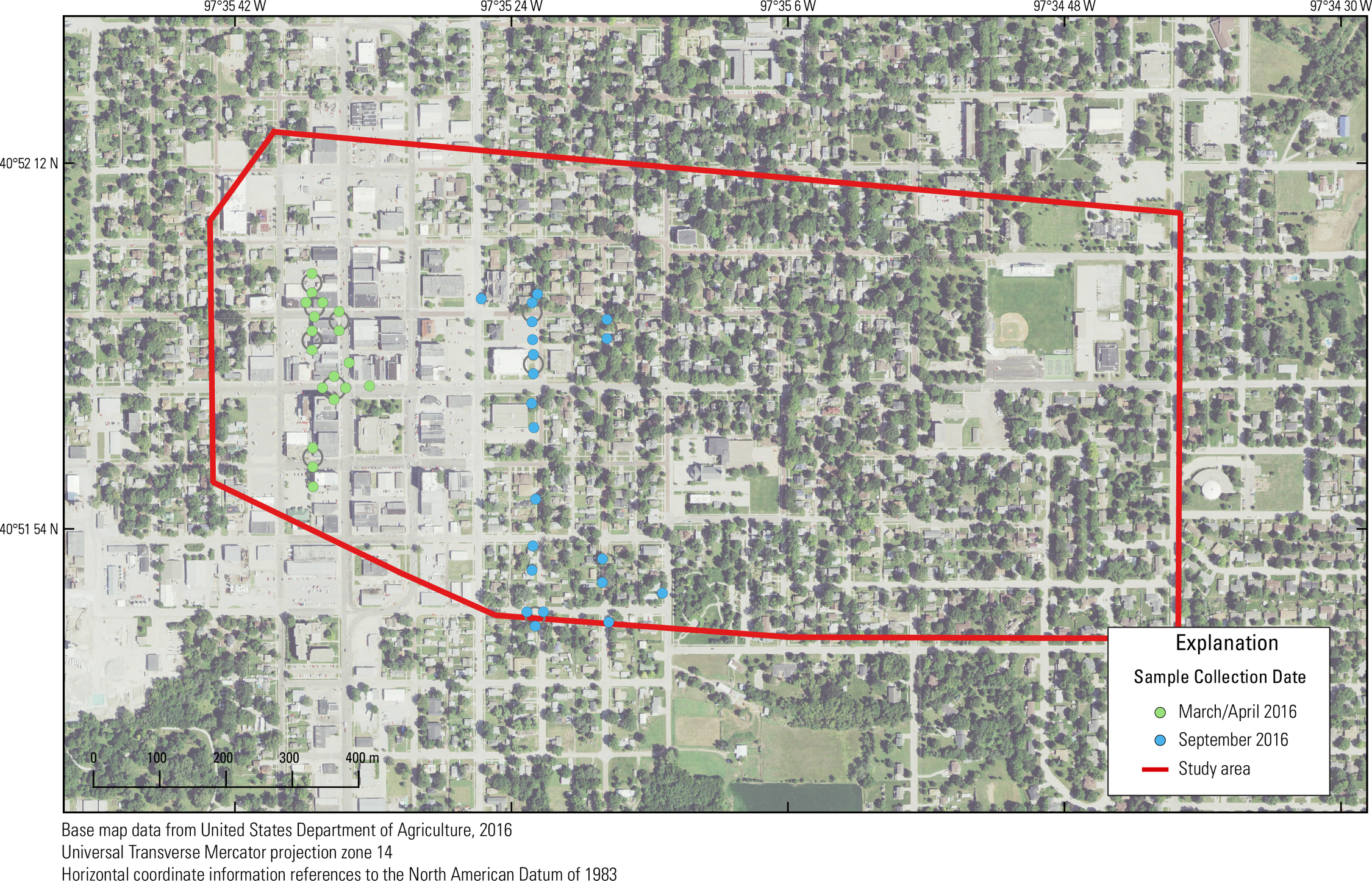

Supplement: S2 Fig — Each set of points in concentric rings represents multiple samples in one area. (TIF) [file pone.0193247.s002.tif]

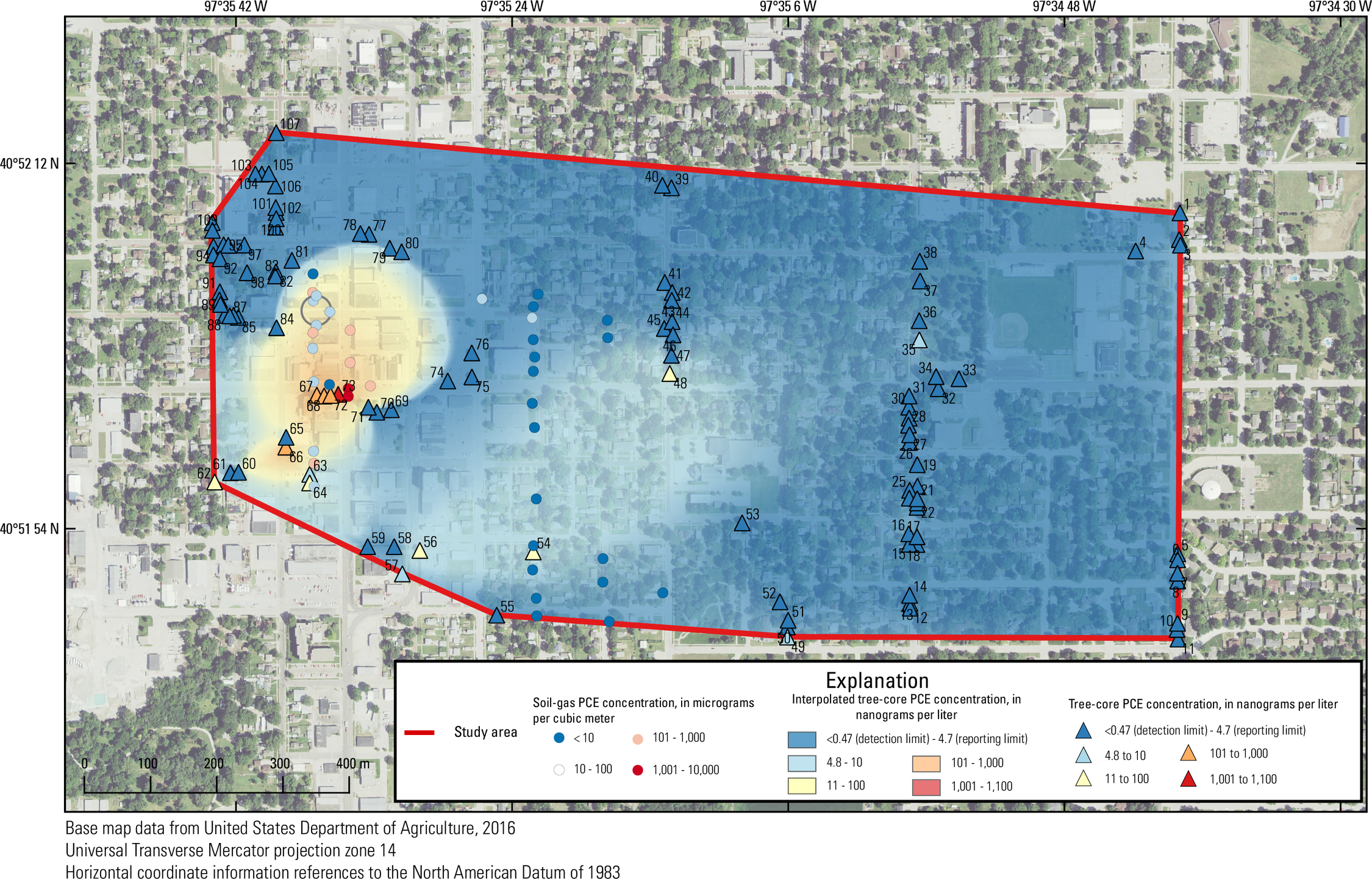

Supplement: S3 Fig — Each set of points in concentric rings represents multiple samples in one area. (TIF) [file pone.0193247.s003.tif]

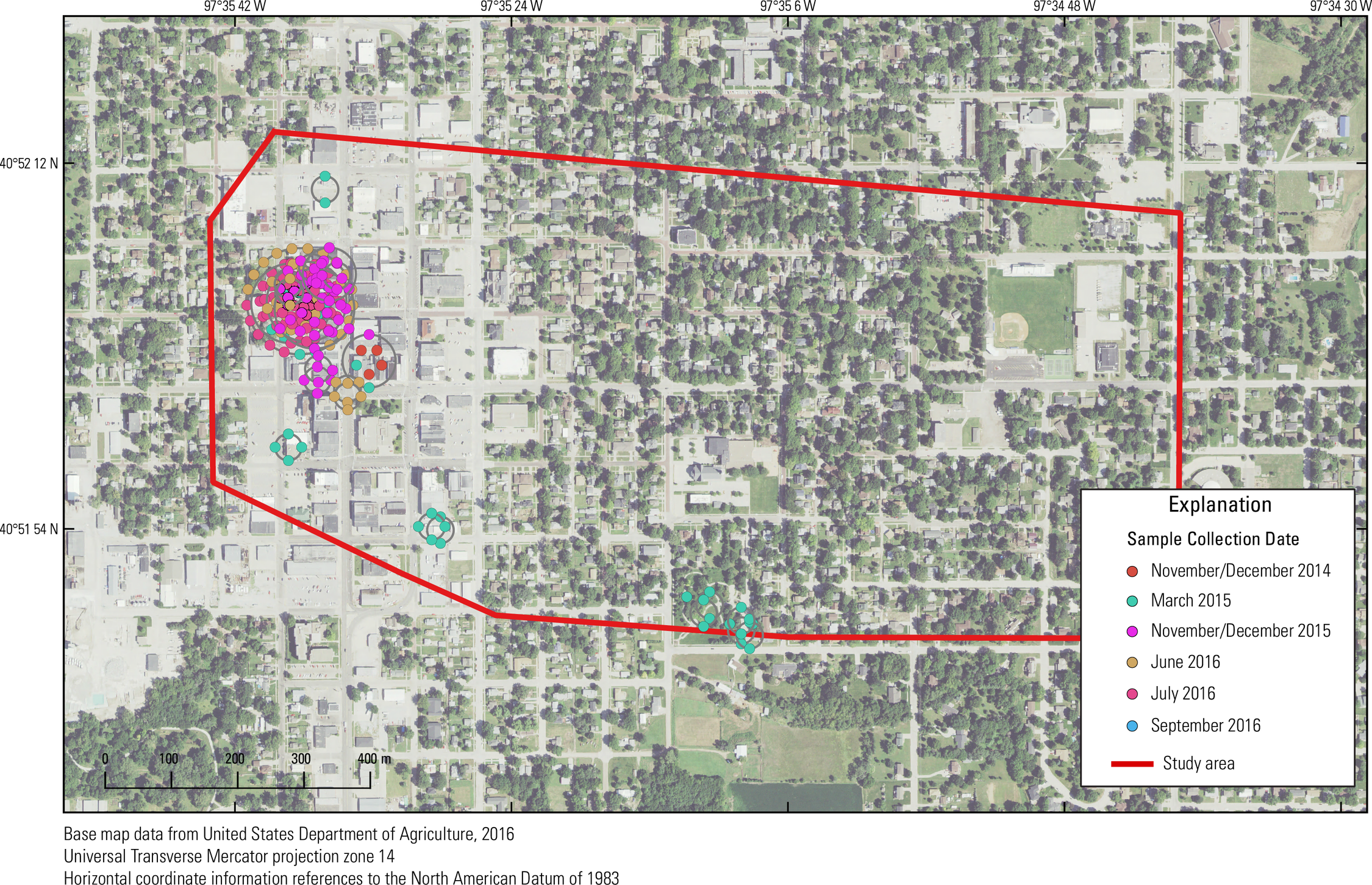

Supplement: S4 Fig — Each set of points in concentric rings represents multiple samples in one area. (TIF) [file pone.0193247.s004.tif]

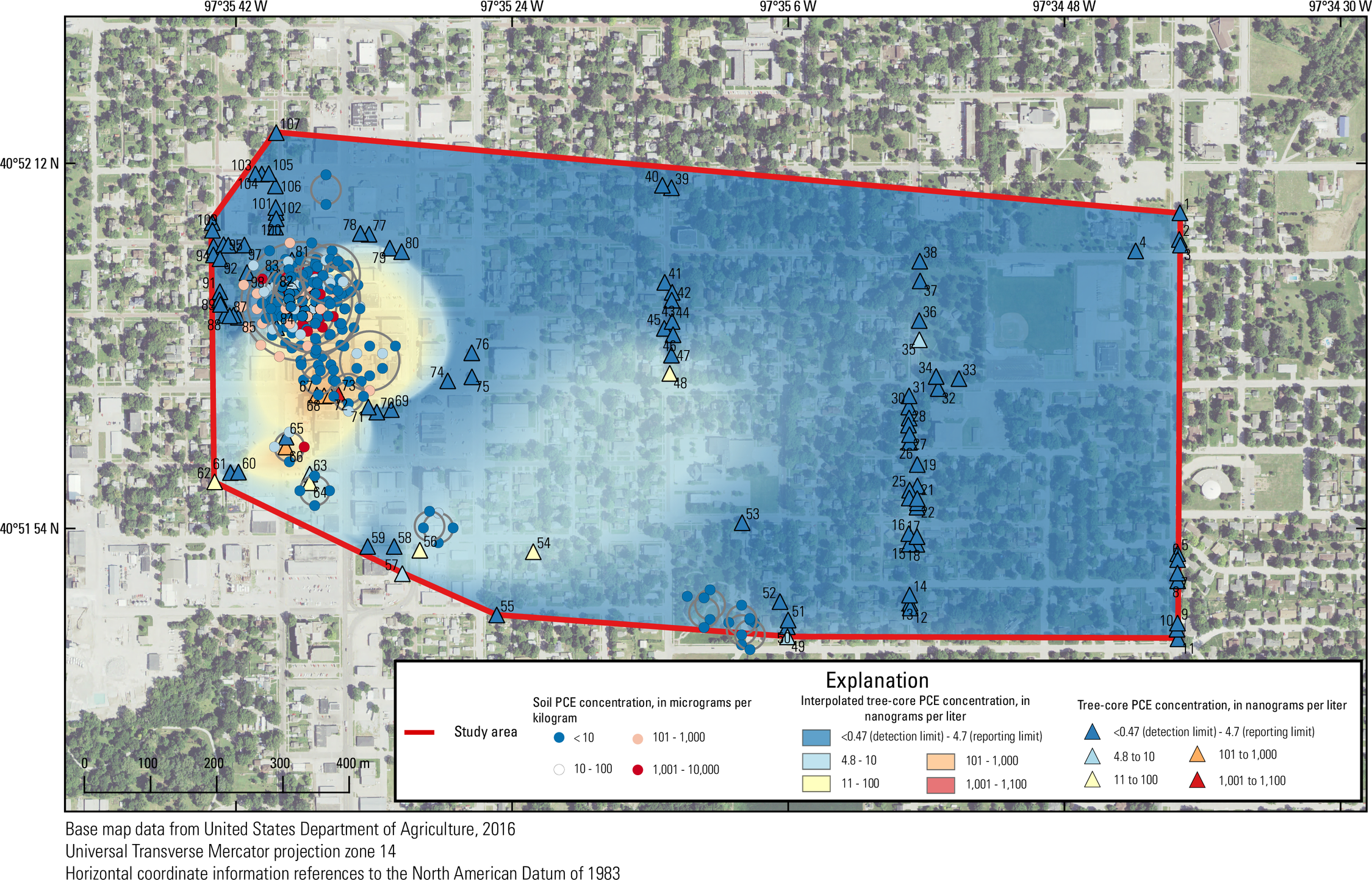

Supplement: S5 Fig — Each set of points in concentric rings represents multiple samples in one area. (TIF) [file pone.0193247.s005.tif]

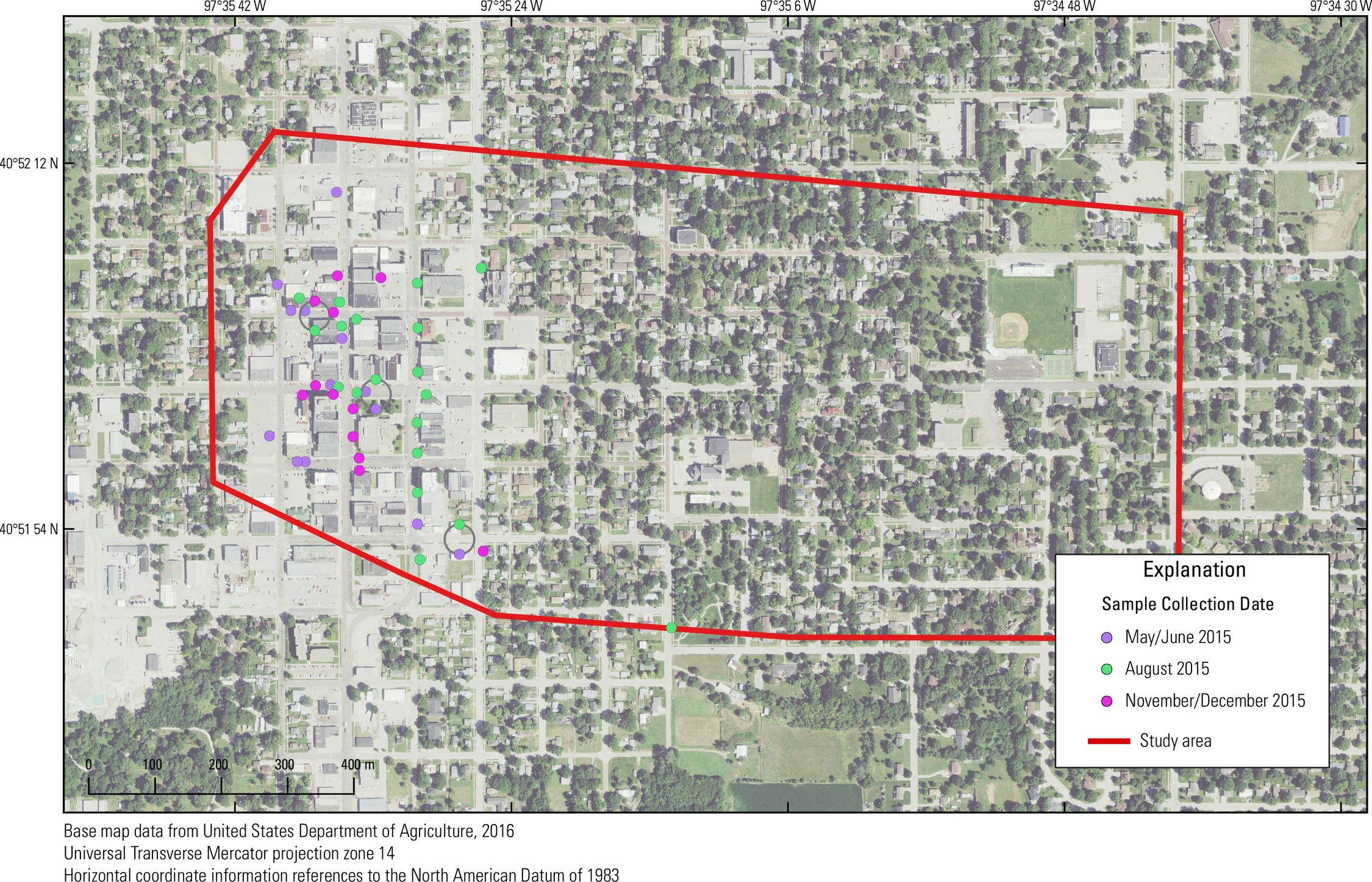

Supplement: S6 Fig — Each set of points in concentric rings represents multiple samples in one area. (TIF) [file pone.0193247.s006.tif]

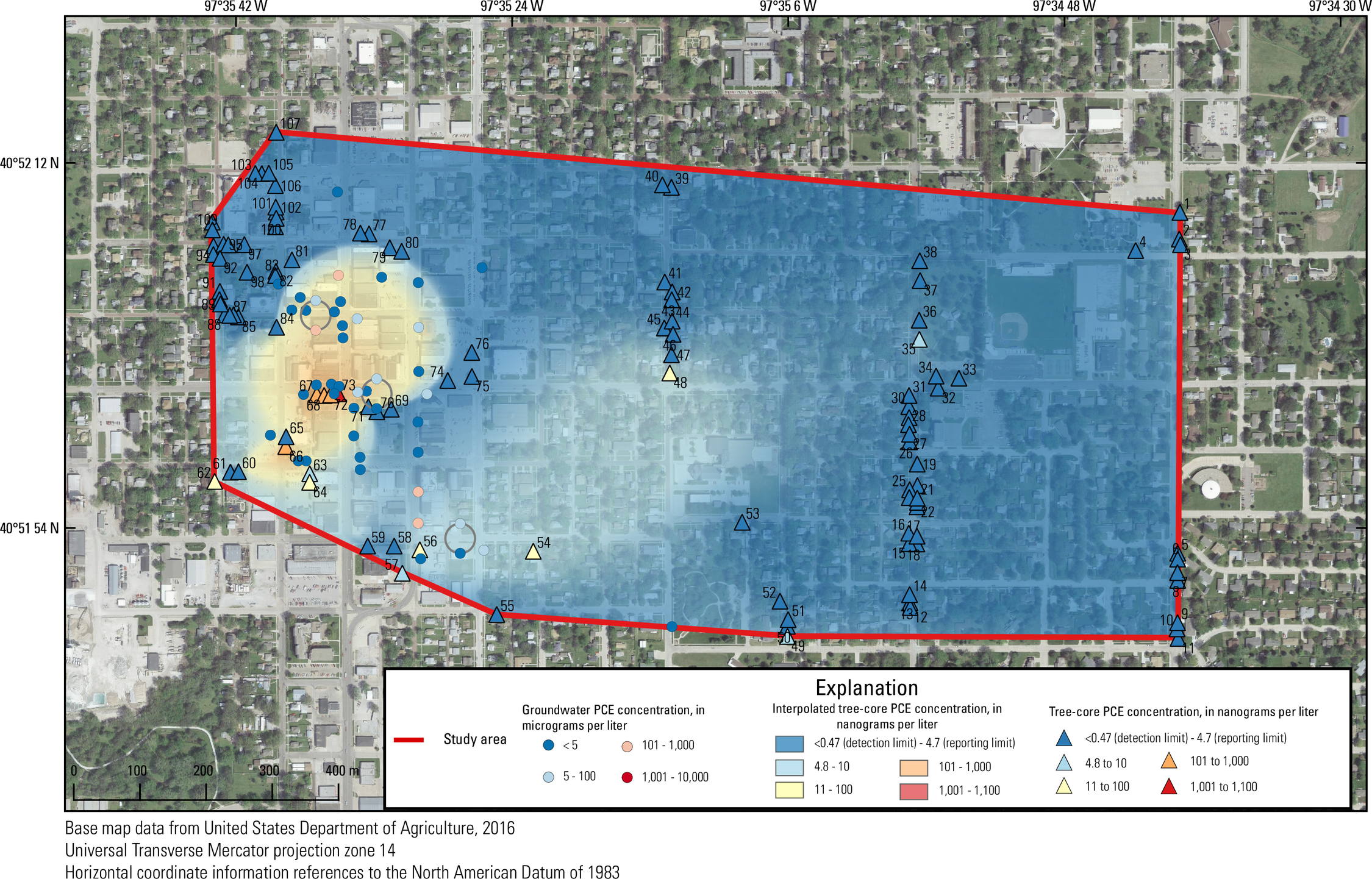

Supplement: S7 Fig — Each set of points in concentric rings represents multiple samples in one area. (TIF) [file pone.0193247.s007.tif]

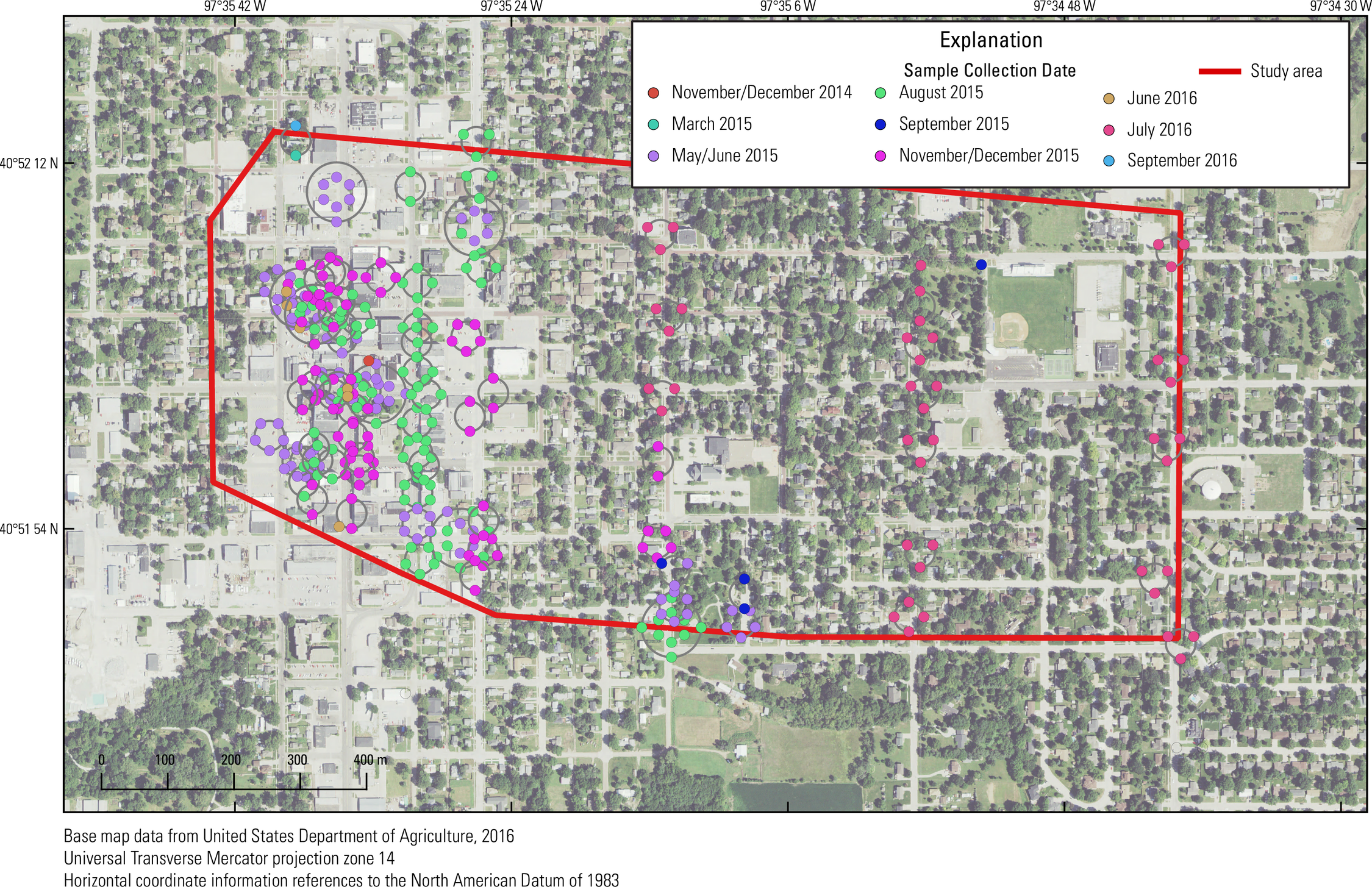

Supplement: S8 Fig — Each set of points in concentric rings represents multiple samples in one area. (TIF) [file pone.0193247.s008.tif]

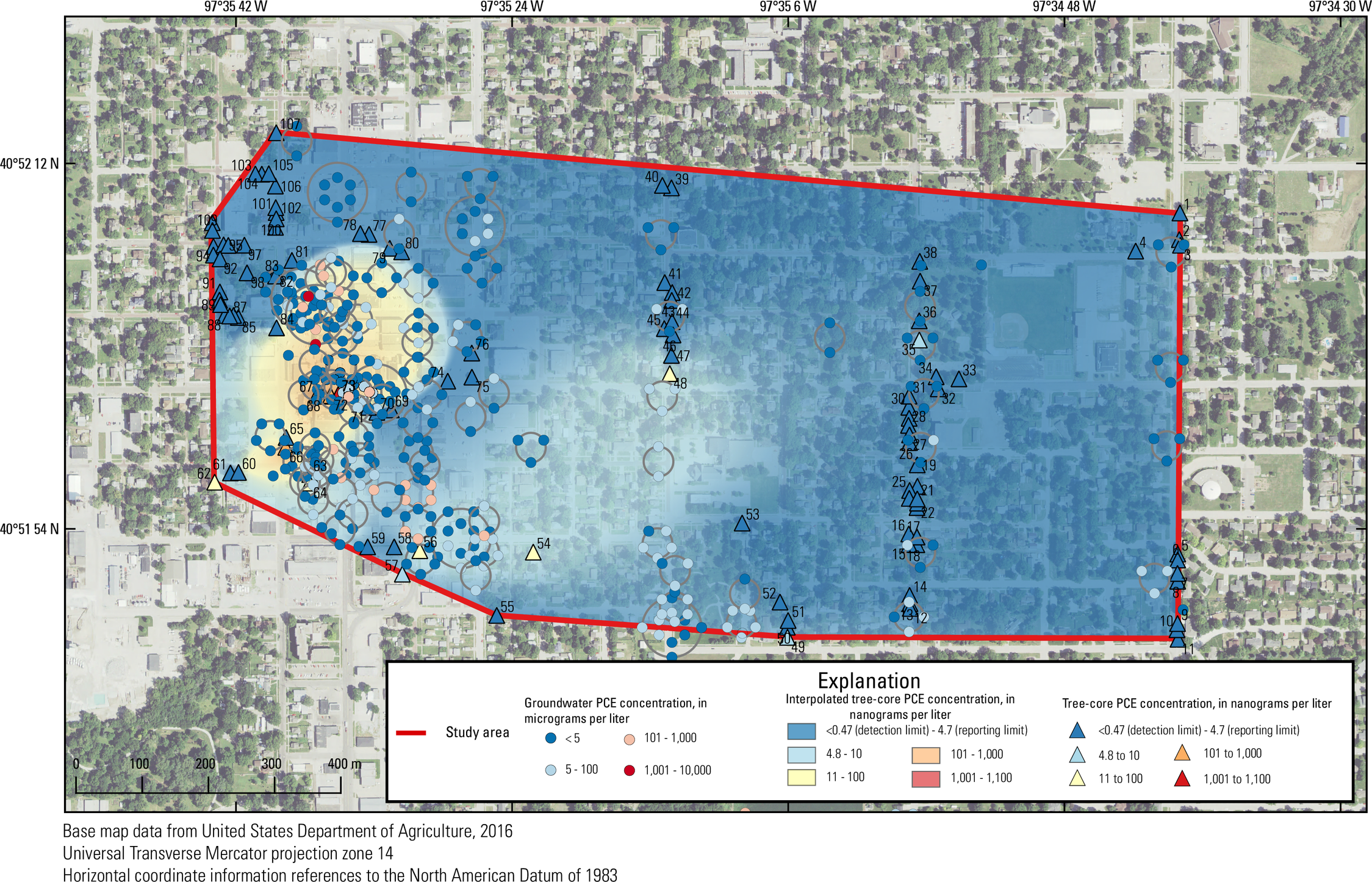

Supplement: S9 Fig — Each set of points in concentric rings represents multiple samples in one area. (TIF) [file pone.0193247.s009.tif]

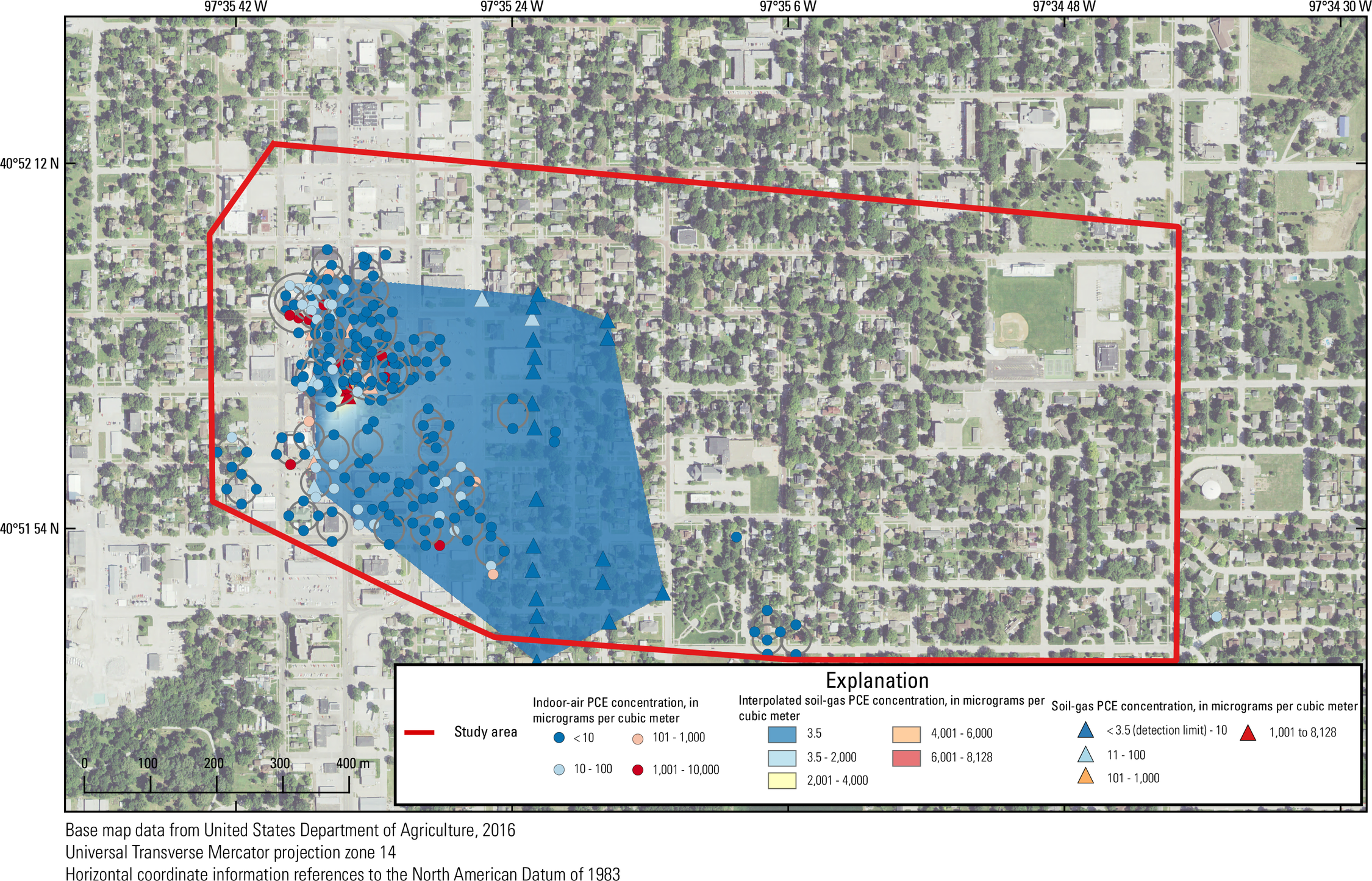

Supplement: S10 Fig — Each set of points in concentric rings represents multiple samples in one area. (TIF) [file pone.0193247.s010.tif]

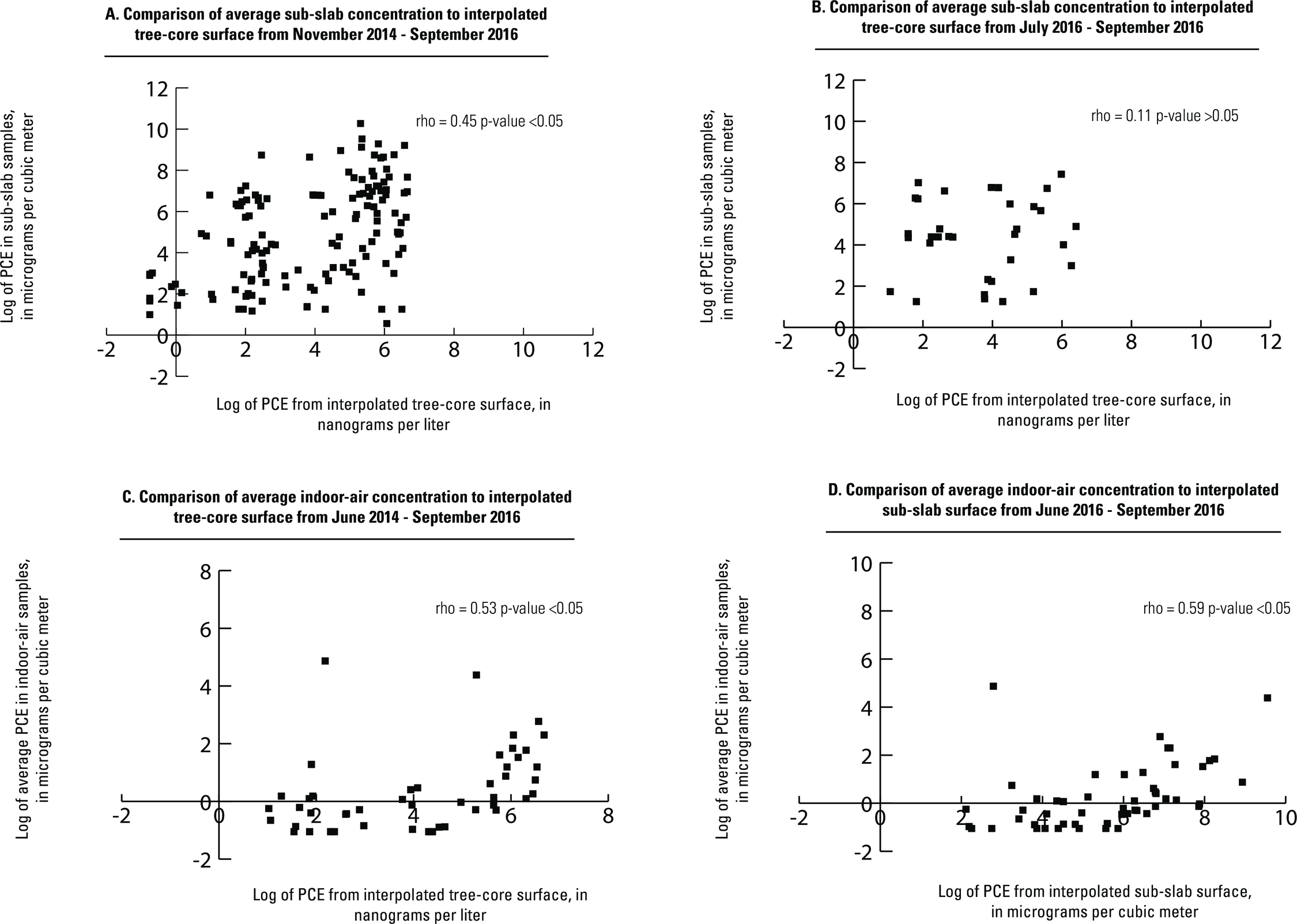

Supplement: S11 Fig — Rho is the spearman’s rank correlation coefficient. (TIF) [file pone.0193247.s011.tif]

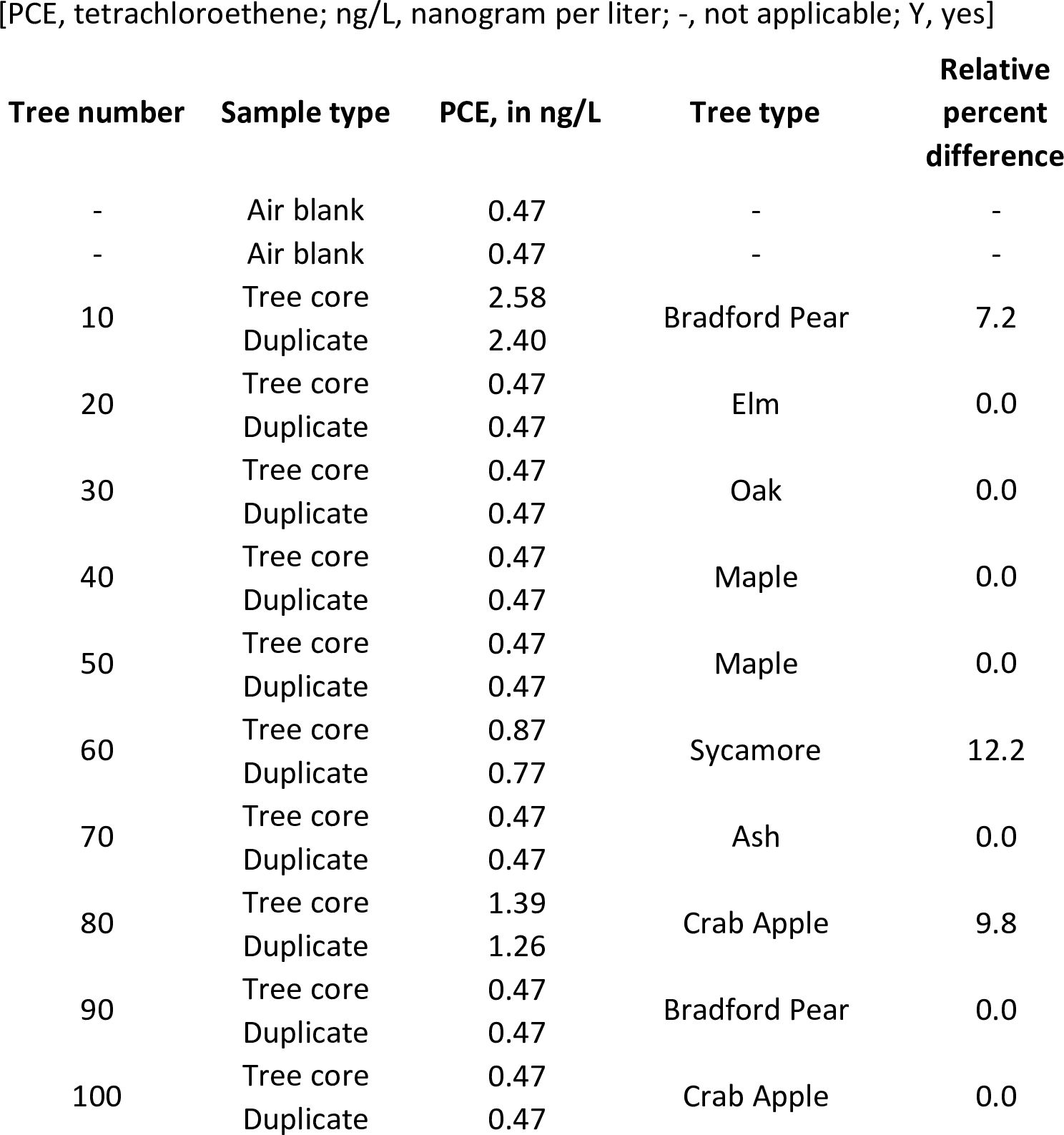

Supplement: S1 Table — (TIF) [file pone.0193247.s012.tif]
